# Causal association of epigenetic aging and osteoporosis: a bidirectional Mendelian randomization study

**DOI:** 10.1186/s12920-023-01708-3

**Published:** 2023-11-02

**Authors:** Xinyu Liang, Wei Shi, Xinglong Zhang, Ran Pang, Kai Zhang, Qian Xu, Chunlei Xu, Xin Wan, Wenhao Cui, Dong Li, Zhaohui Jiang, Zhengxuan Liu, Hui Li, Huafeng Zhang, Zhijun Li

**Affiliations:** 1 Department of Orthopedics, Tianjin Medical University General Hospital, Tianjin, 300052 People’s Republic of China; 2 Department of Orthopedics, Tianjin Hospital of ITCWM Nankai Hospital, Tianjin, People’s Republic of China; 3 School of Integrative Medicine, Tianjin University of Traditional Chinese Medicine, Tianjin, People’s Republic of China; 4 Department of Orthopedics, Tian-Jin Union Medical Centre, Nankai University People’s Hospital, Tianjin, China; 5 Department of Pharmacology, Kyoto Prefectural University of Medicine, Kyoto, Japan; 6 R&D Center, Youjia (Hangzhou) Biomedical Technology Co., Ltd, Hangzhou, China; 7 Department of Orthopaedic, Wenzhou Central Hospital, Wenzhou, China

**Keywords:** Mendelian randomization, Epigenetic age, Osteoporosis, Causal association, Genome-wide association study

## Abstract

**Background:**

The relationship between aging and osteoporosis is well established. However, the relationship between the body's physiological age, i.e. epigenetic age, and osteoporosis is not known. Our goal is to analyze the bidirectional causal relationship between epigenetic clocks and osteoporosis using a bidirectional Mendelian randomization study.

**Methods:**

We used SNPs closely associated with GrimAge, Hannum, PhenoAge, and HorvathAge in epigenetic age and SNPs closely associated with femoral neck bone mineral density, lumbar spine bone mineral density, and forearm bone mineral density as instrumental variables, respectively, using the inverse variance weighting method and several other MR methods to assess the bidirectional causal relationship between epigenetic age and osteoporosis.

**Result:**

There was no evidence of a clear causal relationship of epigenetic age (GrimAge, Hannum, PhenoAge, and HorvathAge) on femoral neck bone mineral density, lumbar spine bone mineral density, and forearm bone mineral density. In reverse Mendelian randomization analysis showed a significant causal effect of lumbar spine bone mineral density on GrimAge: odds ratio (OR) = 0.692, 95% confidence interval (CI) = (0.538–0.890), *p* = 0.004. The results suggest that a decrease in lumbar spine bone mineral density promotes an acceleration of GrimAge.

**Conclusion:**

There was no significant bidirectional causal relationship between epigenetic age and osteoporosis A decrease in lumbar spine bone density may lead to an acceleration of the epigenetic clock "GrimAge". Our study provides partial evidence for a bidirectional causal effect between epigenetic age and Osteoporosis.

**Supplementary Information:**

The online version contains supplementary material available at 10.1186/s12920-023-01708-3.

## Introduction

Osteoporosis (OP) is the most common metabolic bone disease, characterized by reduced bone mineral density (BMD) and deterioration of trabecular architecture, and is capable of causing many skeletal-related diseases, with a serious negative impact on the health of the elderly in particular [1, 2]. With more than 9 million OP-related fractures occurring each year, OP has become a major global public health problem, which imposes a significant clinical and economic burden on society and an increased health cost and disease burden on patients [3]. Age is a very important factor in the reduction of bone mineral density and because of lifestyle habits, physical conditions, etc., older adults are more prone to osteoporotic fractures [4, 5]. In addition to age, BMD in osteoporosis and its prognosis involve many other factors such as genetic factors, gender, obesity, calcium and milk intake, caffeine intake, smoking and physical activity [6, 7]. However, both the current diagnostic technique for OP, dual-energy X-ray absorptiometry (DXA), and pharmacological treatment of OP have limitations, and patients with osteoporosis have poor compliance with pharmacological treatment due to adverse reactions associated with drug side effects, so it is crucial to find other risk factors for OP to optimize the diagnosis, prevention, and treatment of OP [8–11].

Epigenetic age is a heritable indicator of biological aging derived from DNA methylation (DNAm) data, which differs from actual age and is a very promising biomarker of aging that has emerged recently [12]. Accelerated epigenetic age means that the predicted biological age is greater than the actual age, and such populations have an increased risk of death from various causes [13, 14]. "First generation" epigenetic clocks, such as HannumAge and Intrinsic HorvathAge, are derived based on DNAm levels at CpG loci that are closely related to actual age [15, 16]. Hannum et al. analyzed whole blood samples from 656 individuals and identified 71 age-associated CpGs, and HannumAge results were derived by training on these loci [16]. Intrinsic HorvathAge is based on training on 353 age-related CpG loci found in human tissues and cells, and further adjusting blood cell counts [15]. "Second generation" epigenetic clocks, such as PhenoAge and GrimAge, have been developed in recent years to predict age-related morbidity and mortality [17, 18]. PhenoAge integrates data from 513 CpG and nine clinical biomarkers (e.g., albumin, creatinine, serum glucose, C-reactive protein, lymphocyte percentage, etc.) associated with mortality [17]. GrimAge was derived from seven plasma proteins (i.e. cystatin C, leptin, tissue inhibitor metalloproteinases 1, adrenomedullin, beta-2-microglobulin, growth differentiation factor 15, and plasminogen activation inhibitor 1 (PAI-1)) and a DNAm-based estimator of smoking pack-years [18].

Since OP is inextricably linked to aging at real age, is there an equally strong relationship between OP and aging at biological age? Differences in DNA methylation signatures of bone tissue samples and mesenchymal stem cells (MSCs) have been found between patients with fragility fractures and osteoarthritis controls, and accelerated epigenetic aging of chondrocytes has been identified [19, 20]. However, no study has been able to prove whether there is a causal relationship between epigenetic age and osteoporosis. In this study, we used MR analysis methods to analyze the causal relationship between epigenetic age and osteoporosis.

Mendelian randomization (MR) analysis is a method of causal inference using genetic variation as an instrumental variable (IV), thereby testing the causal relationship between exposure factors and disease [21–23]. By using GWAS data for Mendelian randomization, we can understand the relationship between a factor and individual diseases [24, 25]. A systematic review and meta-analysis of Mendelian Randomization studies collated Mendelian randomization studies of BMI and multiple diseases and performed a meta-analysis for each disease to systematically look at the causal relationship between exposure measures and disease risk [26]. Standard MR studies must satisfy three core assumptions: (1) instrumental variables are strongly associated with exposure factors; (2) genetic variables are not associated with any confounding factors affecting the causal relationship between exposure and outcome; (3) instrumental variables can only affect outcome through exposure and are not directly associated with outcome [22, 27]. Therefore, we performed a two-way MR analysis to investigate the association between epigenetic age and OP (measured as BMD).

## Methods

### Research design

**Fig. 1:**
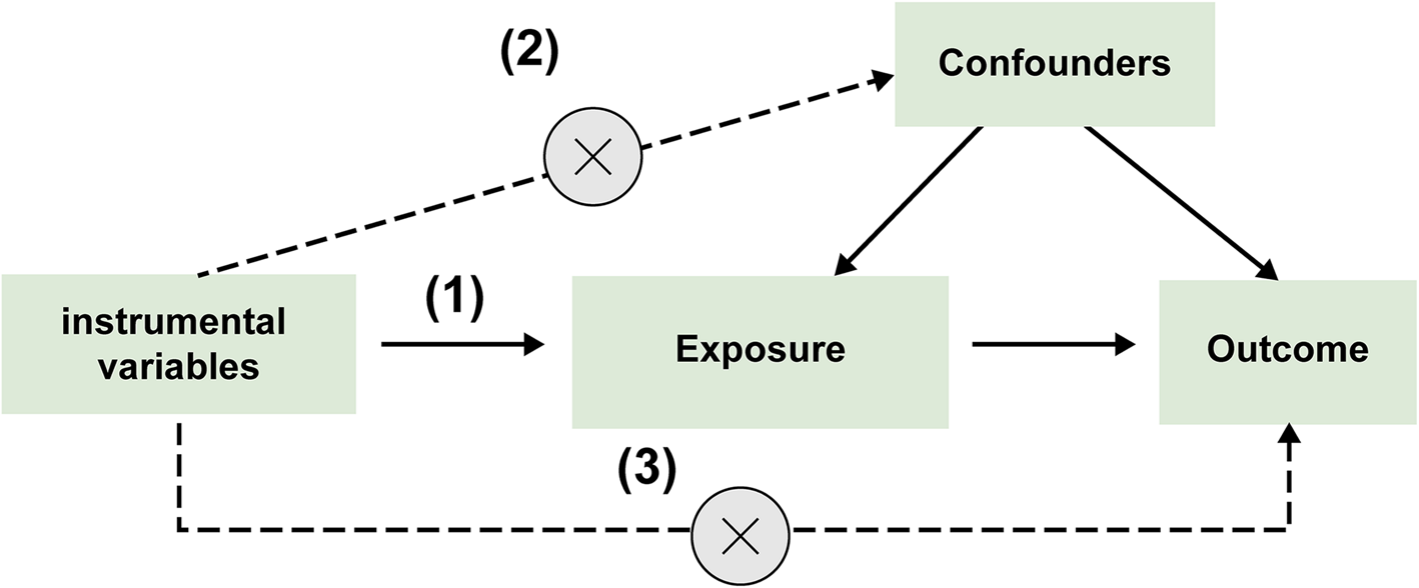
Three major assumptions of Mendelian randomization: (1) instrumental variables are strongly associated with exposure factors; (2) genetic variables are not associated with any confounding factors; and (3) instrumental variables can only influence outcomes through exposure

In this study, we first used epigenetic age (PhenoAge, GrimAge, Hannum, HorvathAge) as the "exposure" and femoral neck BMD (FN BMD), lumbar spine BMD (LS BMD) and forearm BMD (FA BMD) as the "outcome" for Mendelian randomization analysis. In contrast, we used FN BMD, LS BMD and FA BMD as "exposures" and epigenetic age as "outcomes". MR studies consistently meet their three key assumptions: (1) instrumental variables are strongly associated with exposure factors; (2) genetic variables are not associated with any confounding factors affecting the causal relationship between exposure and outcome; and (3) instrumental variables can only influence outcomes through exposure and are not directly associated with outcomes [22, 27]. Figure 1 shows an overview of the three hypotheses.

### Data sources

We obtained summary genetic association estimates for epigenetic age acceleration measures from the recent GWAS biological aging meta-analysis: Intrinsic HorvathAge [15], HannumAge [16], PhenoAge [17] and GrimAge [18]. The analysis was a meta-analysis based on the European ancestry of 34,710 participants from 28 cohorts, 57.3% of whom were women, and it identified 137 loci for DNA biomarkers associated with aging. For more information and a detailed description of the methodology, please see the latest GWAS meta-analysis of biological aging [28].

The diagnosis of OP in clinical practice is often made by BMD at three common skeletal sites: femoral neck BMD (FN BMD), lumbar spine BMD (LS BMD), and forearm BMD (FA BMD). Therefore, we selected the GWAS summary statistics for FA BMD, FN BMD and LS BMD from the GWAS meta-analysis of 53,236 individuals of European ancestry conducted by the Genetic Factors for Osteoporosis (GEFOS) consortium [29]. Relevant GWAS summary statistics are available from the GEFOS website (http://www.gefos.org/?q=content/data-release-2015). Table 1 shows the details of the studies and datasets used for the analysis.

**Table 1:** Description of GWAS data sources

Phenotype	Population	Simple Size	Sex	Access address
GrimAge	Europeans	34,710	Male, Female	https://datashare.ed.ac.uk/handle/10283/3645
Hannum	Europeans	34,710	Male, Female	https://datashare.ed.ac.uk/handle/10283/3645
HorvathAge	Europeans	34,710	Male, Female	https://datashare.ed.ac.uk/handle/10283/3645
PhenoAge	Europeans	34,710	Male, Female	https://datashare.ed.ac.uk/handle/10283/3645
FN BMD	Europeans	32,735	Male, Female	http://www.gefos.org/?q=content/data-release-2015
FA BMD	Europeans	8143	Male, Female	http://www.gefos.org/?q=content/data-release-2015
LS BMD	Europeans	28,498	Male, Female	http://www.gefos.org/?q=content/data-release-2015

### Selection of instrumental variables

Selection of appropriate instrumental variables (IV) for MR analysis from the different GWAS summary results. Independent single nucleotide polymorphisms (SNPs) with significant genome -wide significance (*p* < 5 × 10^–8^) associated with the exposure were selected as IV. However, only three SNPs were selected for FA BMD when screening instrumental variables from GWAS data for FA BMD, so we adjusted the threshold (*p* < 5 × 10^–6^) in order to obtain a relatively appropriate number of instrumental variables. Set the parameter r^2^ threshold to 0.001 and kilobase pairs (kb) to 10,000, and exclude the linkage disequilibrium (LD) by the clump_data function in the "TwoSampleMR" R package. The last valid SNPs obtained that were significantly associated with exposure were used as IV. In this study, we calculated the F values by which the intensity of the screened IVs was assessed [30]. F value of a single SNP = beta^2^/SE^2^ [31]. IVs with F values > 10 have good instrumental strength and can mitigate the potential bias present in MR analysis. The proportions of trait variance explained by genetic instruments (R^2^) is calculated by the following equation: R^2^ = 2 × MAF × (1-MAF) × beta (MAF = minor allele frequency, beta = effect size, SE = standard error, N = sample size, k = number of IVs) [32]. In order to verify whether the selected instrumental variables satisfied the independence hypothesis, PhenoScanner (http://www.phenoscanner.medschl.cam.ac.uk/) was used to detect whether the remaining SNPs were correlated with other phenotypes, such as basal metabolic rate and rheumatoid arthritis, and the results showed that no SNPs was significantly associated with these factors [33, 34].

### Statistical analysis

The inverse variance weighting (IVW) method uses meta-analysis methods to combine the Wald estimates for each SNP to assess the effect of exposure on outcome. The IVW method was used as the primary statistical method to assess the bidirectional relationship between epigenetic age and OP. The IVW method provides unbiased effect estimates when the selected IVs are all valid IVs (when there is no horizontal pleiotropy or heterogeneity). We also performed MR-Egger method, weighted median method (WME), simple mode and weighted mode to complement the MR results. The WME method produces valid causal estimates when the valid IVs of all selected IVs are greater than 50% [35]. We also calculated causal effect estimates (equivalent to beta coefficients) and converted them into dominance ratios (OR).

A series of sensitivity analyses were conducted for the accuracy and robustness of the results. Heterogeneity between IVs was assessed by Cochran Q statistic [36]. If there was heterogeneity in the data, we removed the SNPs causing heterogeneity by MR-PRESSO analysis(with P value less than the threshold in the MR-PRESSO outlier test). If the heterogeneity was still significant after removing the abnormal values by MR-PRESSO, we would perform MR analysis again under the condition of removing all abnormal SNPs with P values less than 1 in MR-PRESSO [37]. We assessed horizontal pleiotropy by analyzing MR Egger intercept values [27]. Then, we analyzed each SNP by using the "leave-one-out" method, and checked whether there was a high effect on the effect estimates by eliminating each SNP one by one.

The above statistical treatments were implemented by software R4.2.2 and TwoSampleMR package.

## Results

### Instrumental variable

We screened 4, 9, 24, and 11 IVs from the GWAS data of GrimAge, HannumAge, Intrinsic HorvathAge, and PhenoAge, respectively, and the F statistics of all selected SNPs were greater than 10 (GrimAge range 31–45, HannumAge range 31–99, Intrinsic HorvathAge range 31–240, PhenoAge range 32–89) (Supplementary Table 1). 20, 16, 22 IVs were screened from the GWAS data of FN BMD,FA BMD, LS BMD. All selected SNPs had F statistics greater than 10 (FN BMD range 32–111, FA BMD range 22–114, LS BMD range 32–89) (Supplementary Table 2). The instrument strength in this study is high and the results of MR analysis are not affected by weak instrumental variable bias.

### Bidirectional Mendelian randomization results

**Fig. 2:**
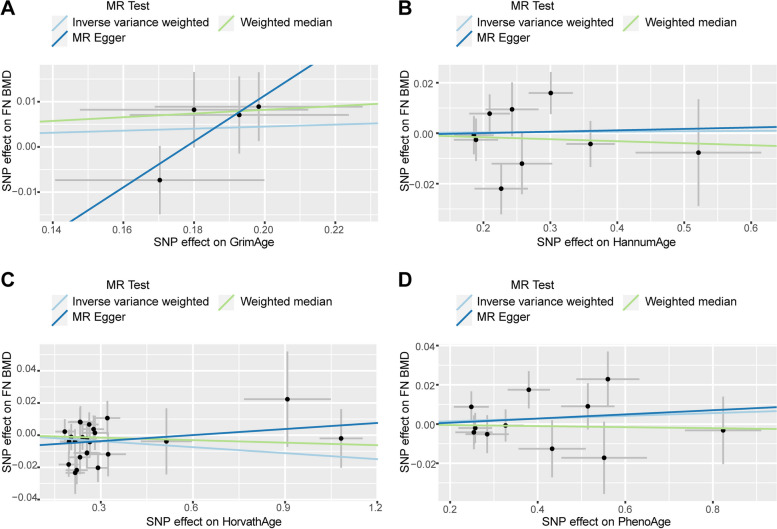
Scatter plot of causality analysis of epigenetic age on FN BMD. Four epigenetic clocks as exposure and FN BMD as outcome. **A** GrimAge **B** Hannum **C** HorvathAge **D** PhenoAge

**Fig. 3:**
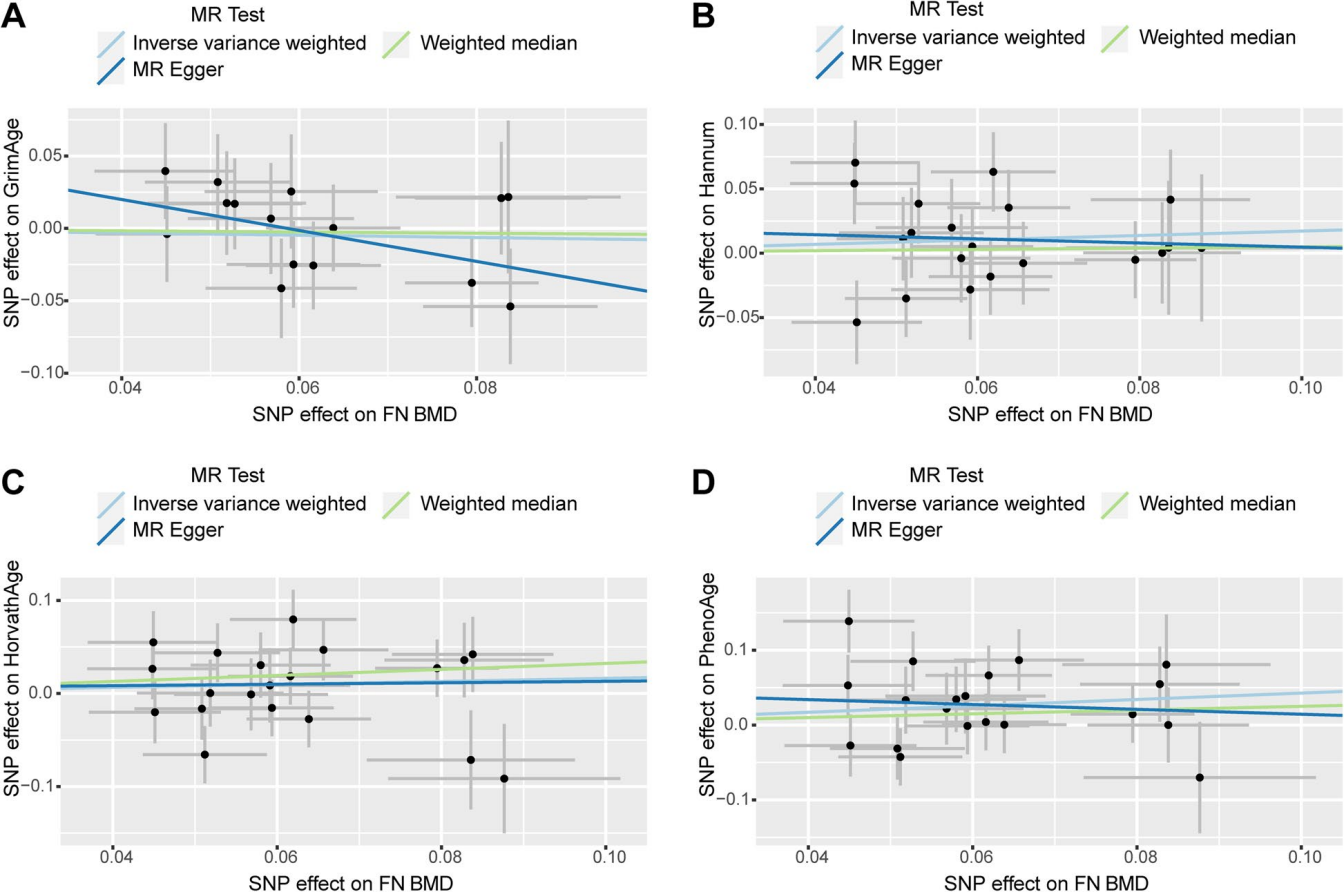
Scatter plot of causality analysis of FN BMD on epigenetic age. FN BMD as exposure and four epigenetic clocks as outcome. **A** GrimAge **B** Hannum **C** HorvathAge **D** PhenoAge

We observed no causal relationship between epigenetic age and BMD in Mendelian randomization analysis. In the causal relationship analysis of the epigenetic clock on FN BMD, we calculated the OR and 95% CI: GrimAge and FN BMD: 1.023 (95%CI 0.980–1.067),*P* = 0.297;HannumAge and FN BMD: 1.002(95%CI 0.973–1.031), *P* = 0.915; Intrinsic HorvathAge and FN BMD: 0.988(95%CI 0.973–1.002), P = 0.100; PhenoAge and FN BMD: 1.007(95%CI 0.991–1.023), *P* = 0.401. Therefore, we found that there was no statistically significant (Table 2). For detailed information on the MR analysis of the causal relationship of the epigenetic clock on BMD refer to Supplementary Table 3. The scatter plot is shown in Fig. 2. When performing reverse MR (exposure: FN BMD, outcome: epigenetic age), we found that the IVW results of FN BMD and PhenoAge showed a causal relationship between the two. (beta = 0.427, SE = 0.181,*p* = 0.019). But in the MR Egger method the result is: beta = -0.325,SE = 0.929,*p* = 0.730 (Table 3). It can be found that the beta values of the two methods are not in the same direction, indicating that the results obtained are not robust, so a clear causal relationship between the two cannot be established yet. It can be found by other methods that only the results of IVW are more significant, while all other methods are not significant, and on the whole, it cannot be considered that there is a causal relationship between them. Additional analysis methods can be found in Supplementary Table 4. The scatter plot is shown in Fig. 3. The pleiotropy analysis showed no pleiotropy in the results. Heterogeneity analysis revealed a heterogeneity between FN and GrimAge, so we performed MR-PRESSO analysis. After removing the abnormal IV, the heterogeneity disappeared, and MR analysis was performed again, which resulted in negative results (Table 4).

**Table 2:** Mendelian randomization analysis for epigenetic aging on BMD

Exposure	Outcome	Method	nSNP	Beta	SE	P	OR
GrimAge	FN-BMD	IVW	4	0.022	0.021	0.297	1.023(0.980–1.067)
GrimAge	FA-BMD	IVW	4	-0.026	0.045	0.569	0.975(0.893–1.064)
GrimAge	LS-BMD	IVW	4	-0.036	0.025	0.151	0.965(0.919–1.013)
HannumAge	FN-BMD	IVW	9	0.002	0.015	0.915	1.002(0.973–1.031)
HannumAge	FA-BMD	IVW	9	0.028	0.025	0.259	1.029(0.979–1.081)
HannumAge	LS-BMD	IVW	8	0.008	0.021	0.681	1.008(0.969–1.050)
HorvathAge	FN-BMD	IVW	24	-0.012	0.008	0.100	0.988(0.973–1.002)
HorvathAge	FA-BMD	IVW	24	-0.009	0.017	0.579	0.991(0.958–1.024)
HorvathAge	LS-BMD	IVW	24	-0.013	0.008	0.114	0.987(0.972–1.003)
PhenoAge	FN-BMD	IVW	11	0.007	0.008	0.401	1.007(0.991–1.023)
PhenoAge	FA-BMD	IVW	11	0.011	0.172	0.534	1.011(0.977–1.045)
PhenoAge	LS-BMD	IVW	11	-0.001	0.010	0.926	0.999(0.980–1.018)

**Table 3:** Mendelian randomization analysis for BMD on epigenetic aging

Exposure	Outcome	Method	nSNP	Beta	SE	P	OR
FN-BMD	GrimAge	IVW	15	-0.079	0.143	0.582	0.924(0.699–1.223)
FN-BMD	HannumAge	IVW	20	0.172	0.125	0.169	1.188(0.929–1.519)
FN-BMD	HorvathAge	IVW	20	0.162	0.149	0.275	1.176(0.879–1.574)
FN-BMD	PhenoAge	IVW	20	0.427	0.181	0.019	1.532(1.074–2.186)
FN-BMD	PhenoAge	MR Egger	20	-0.325	0.928	0.730	0.723(0.117–4.458)
FA-BMD	GrimAge	IVW	16	-0.140	0.113	0.217	0.870(0.697–1.085)
FA-BMD	HannumAge	IVW	16	-0.059	0.101	0.557	0.943(0.774–1.148)
FA-BMD	HorvathAge	IVW	16	0.108	0.093	0.248	1.114(0.928–1.337)
FA-BMD	PhenoAge	IVW	16	0.006	0.117	0.960	1.006(0.799–1.266)
LS-BMD	GrimAge	IVW	17	-0.368	0.129	0.004	0.692(0.538–0.890)
LS-BMD	HannumAge	IVW	18	-0.058	0.128	0.650	0.944(0.734–1.212)
LS-BMD	HorvathAge	IVW	22	0.088	0.132	0.503	1.092(0.844–1.415)
LS-BMD	PhenoAge	IVW	22	-0.060	0.157	0.704	0.942(0.692–1.282)

**Table 4:** Heterogeneity and pleiotropic analysis for epigenetic aging and BMD after removing all the SNPs

Exposure	Outcome	Heterogeneity analysis		Pleiotropic test	
		Q	*P* value	intercept	*P* value
GrimAge	FN-BMD	2.881	0.410	-0.090	0.302
GrimAge	FA-BMD	2.279	0.517	0.076	0.634
GrimAge	LS-BMD	1.226	0.747	-0.070	0.454
HannumAge	FN-BMD	11.563	0.172	-0.001	0.944
HannumAge	FA-BMD	6.593	0.581	-0.008	0.755
HannumAge	LS-BMD	13.523	0.060	-0.006	0.774
HorvathAge	FN-BMD	27.523	0.234	-0.007	0.173
HorvathAge	FA-BMD	33.045	0.080	-0.004	0.754
HorvathAge	LS-BMD	19.754	0.657	-0.005	0.359
PhenoAge	FN-BMD	9.421	0.493	-0.001	0.873
PhenoAge	FA-BMD	5.041	0.888	0.017	0.369
PhenoAge	LS-BMD	5.029	0.889	-0.007	0.526
FN-BMD	GrimAge	9.735	0.781	0.063	0.185
FN-BMD	HannumAge	19.713	0.412	0.021	0.606
FN-BMD	HorvathAge	26.191	0.125	0.005	0.912
FN-BMD	PhenoAge	25.049	0.159	0.047	0.420
FA-BMD	GrimAge	22.460	0.096	0.008	0.816
FA-BMD	HannumAge	18.566	0.234	-0.015	0.618
FA-BMD	HorvathAge	10.340	0.798	-0.045	0.120
FA-BMD	PhenoAge	7.826	0.931	-0.018	0.606
LS-BMD	GrimAge	12.083	0.738	0.006	0.871
LS-BMD	HannumAge	17.953	0.392	-0.030	0.390
LS-BMD	HorvathAge	30.036	0.091	-0.020	0.625
LS-BMD	PhenoAge	27.207	0.164	-0.017	0.729

**Fig. 4:**
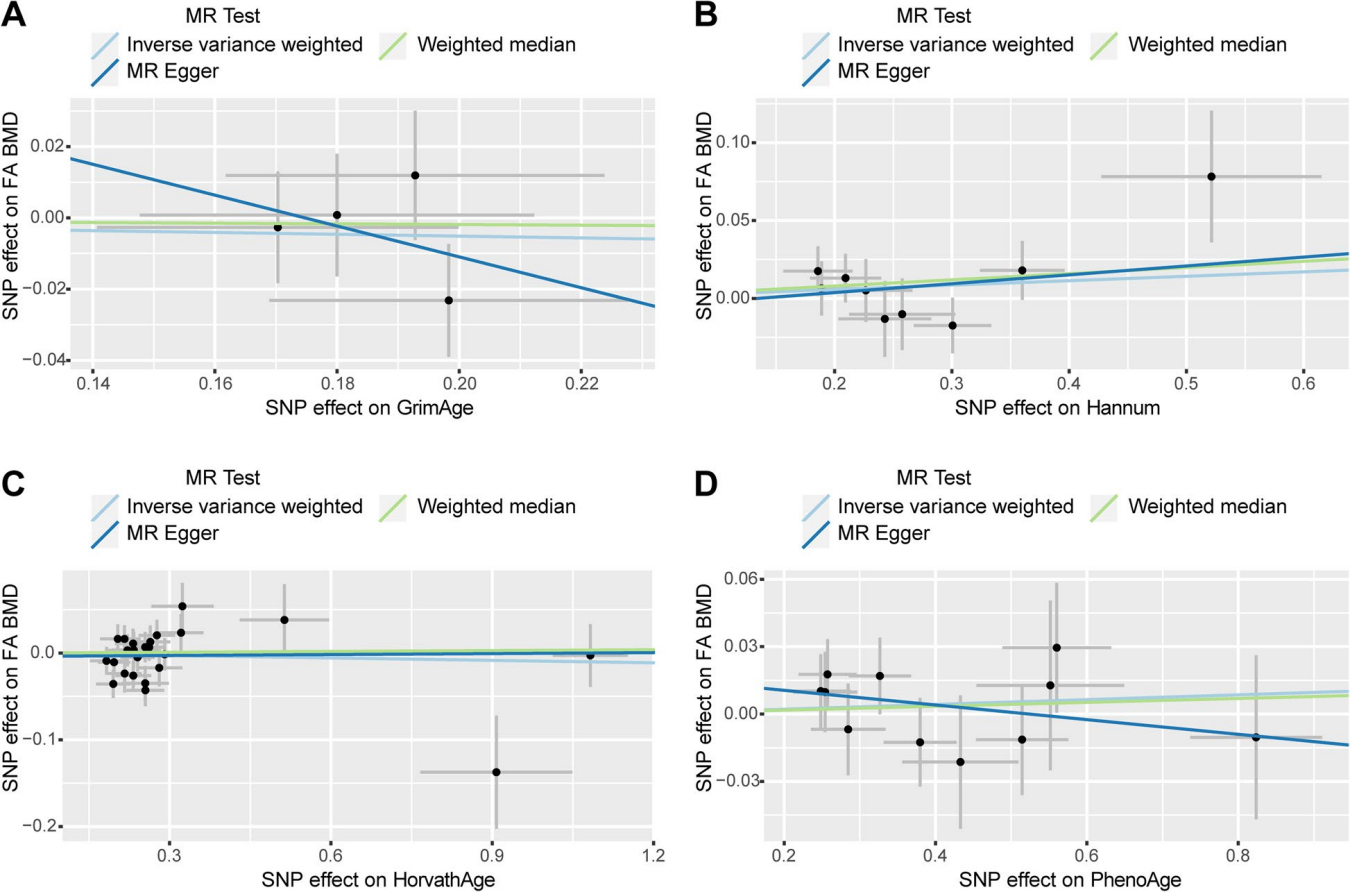
Scatter plot of causality analysis of epigenetic age on FA BMD. Four epigenetic clocks as exposure and FA BMD as outcome. **A** GrimAge **B** Hannum **C** HorvathAge **D** PhenoAge

**Fig. 5:**
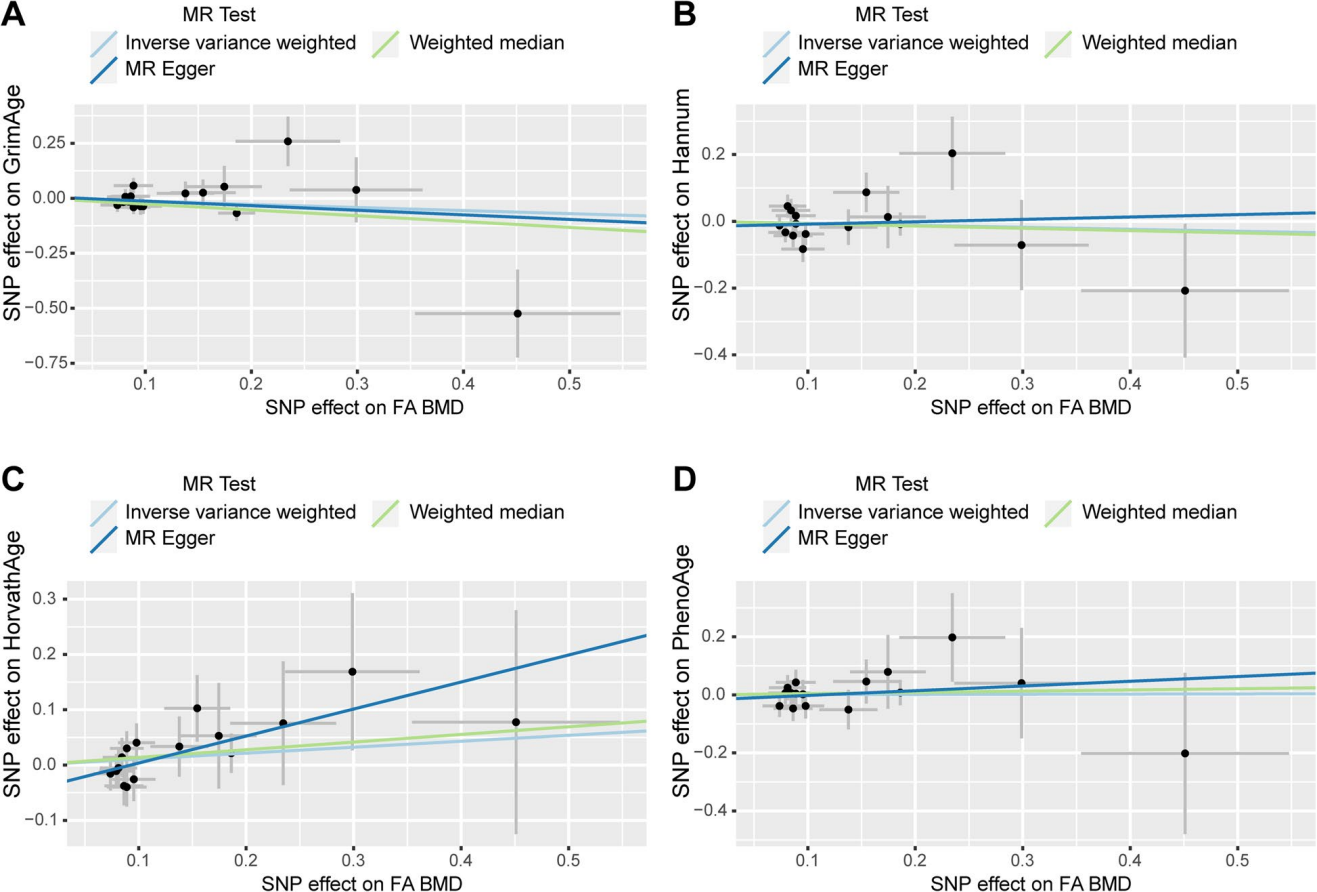
Scatter plot of causality analysis of FA BMD on epigenetic age. FA BMD as exposure and four epigenetic clocks as outcome. **A** GrimAge **B** Hannum **C** HorvathAge **D** PhenoAge

In the causal relationship analysis of the epigenetic clock on FA BMD, we calculated the OR and 95% CI: GrimAge and FA BMD: 0.975 (95%CI 0.893–1.064),*P* = 0.569;HannumAge and FA BMD: 1.029 (95%CI 0.979–1.081), *P* = 0.259;Intrinsic HorvathAge and FA BMD:0.991(95%CI 0.958–1.024), *P* = 0.579; PhenoAge and FAN BMD:1.011(95%CI 0.977–1.045), *P* = 0.534. We found no statistical significance (Table 4) (Supplementary Table 3). The scatter plot is shown in Fig. 4. In reverse MR analysis (exposure: FA BMD, outcome: epigenetic age), the result is: FA BMD and GrimAge: 0.870(95%CI 0.697–1.085), *P* = 0.217; FA BMD and HannumAge: 0.943(95%CI 0.774–1.148), *P* = 0.557; FA BMD and Intrinsic HorvathAge: 1.114 (95%CI 0.928–1.337), *P* = 0.248; FAN BMD and PhenoAge: 1.006(95%CI 0.799–1.266), *P* = 0.960. The results are not statistically significant (Table 3) (Supplementary Table 4). The results are not heterogeneous or pleiotropic (Table 4). The scatter plot is shown in Fig. 5. Therefore, the results of the bidirectional Mendelian analysis between epigenetic clock and FA BMD concluded that there is no causal relationship between them.

**Fig. 6:**
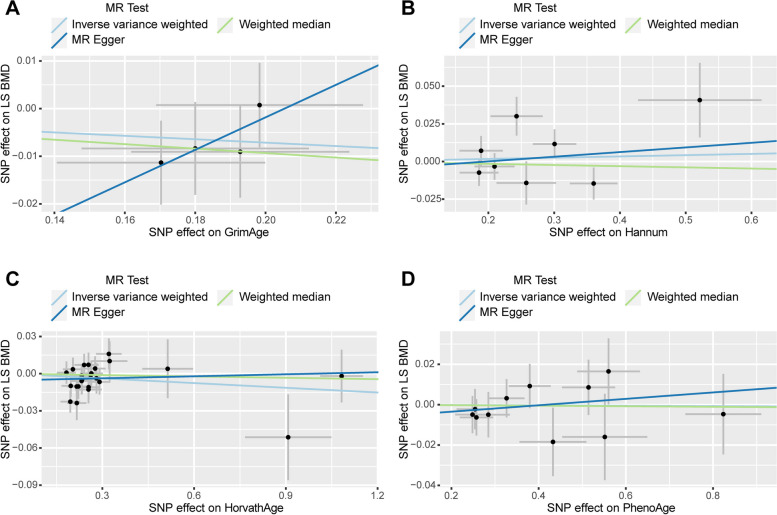
Scatter plot of causality analysis of epigenetic age on LS BMD. Four epigenetic clocks as exposure and LS BMD as outcome. **A** GrimAge **B** Hannum **C** HorvathAge **D** PhenoAge

**Fig. 7:**
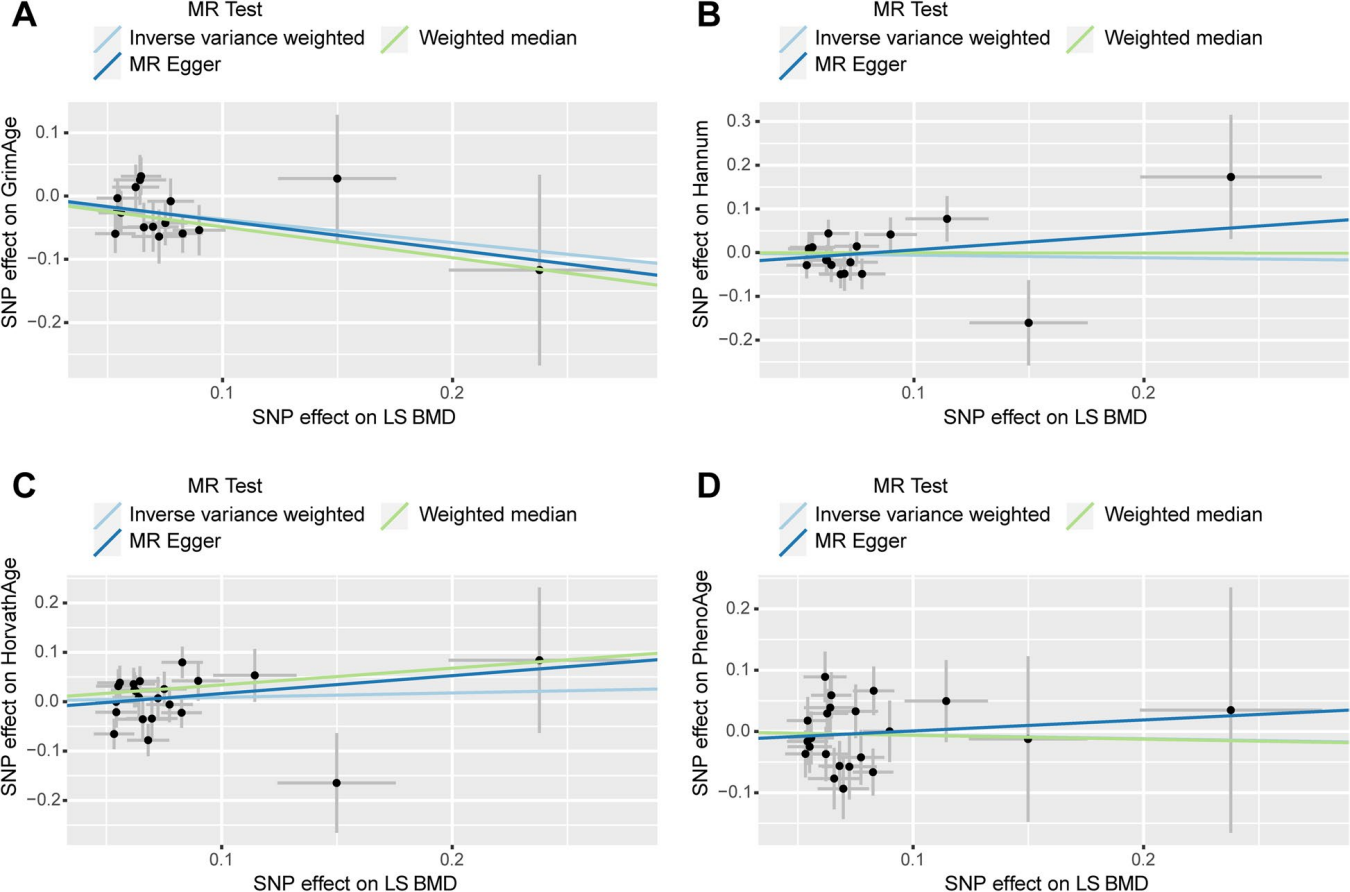
Scatter plot of causality analysis of LS BMD on epigenetic age. LS BMD as exposure and four epigenetic clocks as outcome. **A** GrimAge **B** Hannum **C** HorvathAge **D** PhenoAge

In the causal analysis of the epigenetic clock on LS BMD, we did not find a significant causal relationship between the two. Heterogeneity was found between Hannum and LS BMD when heterogeneity analysis was performed. The heterogeneity disappeared after removal of the abnormal IV (Table 4). The results showed no causal relationship between the two (Table 2) (SupplementaryTable 3). The scatter plot is shown in Fig. 6. When reverse MR analysis was performed (exposure: LS BMD, outcome: epigenetic age), due to the heterogeneity of the results of the two groups, LS BMD and GrimAge, and LS BMD and Hannum, we removed the abnormal IVs in both groups after MR-PRESSO analysis, and the heterogeneity disappeared after removal, and the information after removal of heterogeneity is presented in Table 4. We found a significant causal relationship of LS BMD on GrimAge, with the rest being negative results (Table 3) (Supplementary Table 4). There was no horizontal pleiotropy in the results (Table 4). The scatter plot is shown in Fig. 7.

### Sensitivity analysis

Sensitivity analysis of the IVW method results was performed using the leave-one-out method. The results of “leave-one-out” analysis are shown in Supplementary Figure. Removing one SNP in turn and analyzing the remaining SNPs, we found that the absence of SNPs had a significant impact on the results and the results had significant confidence.

## Discussion

Aging at real age is currently considered to be an important factor in OP, but biological aging is still a potential area for further study. If there is a potential causal relationship between accelerated biological aging and OP, then slowing down biological aging has become a new research direction for preventing OP. In recent years, research between epigenetic mechanisms and osteoporosis is gradually developing, and researchers believe that there is an inextricable relationship between epigenetics and osteoporosis [38]. In our observation of the causal relationship between epigenetic clock and OP, based on our MR results, we found no clear causal relationship of epigenetic clock on osteoporosis. And in the reverse MR analysis, we also did not find an association between the two.We found that the epigenetic clock did not show a clear effect on osteoporosis. In another study of the association between epigenetic age and osteoporosis, investigators analyzed genome-wide DNA profiles of peripheral blood from patients with apparent OP and non-OP controls, but did not find CpG sites with significantly abnormal DNAm in OP, and did not find epigenetic age acceleration in the blood of patients with OP [39].

In this study, we tried to investigate whether osteoporosis could accelerate or delay epigenetic age by reverse Mendelian randomization analysis. We found that LS-BMD has a negative causal relationship to the GrimAge clock of epigenetic age, i.e., a reduction in lumbar spine bone mineral density promotes acceleration of epigenetic age (GrimAge). According to our results, among the four epigenetic clocks, it was found that LS-BMD only showed a significant causal relationship with the GrimAge clock, but this result was not meaningless. Different clocks capture different processes of biological aging, so different biological mechanisms of aging can be understood [12].

GrimAge is better able to predict age-related clinical phenotypes and all-cause mortality than the other three clocks [40]. Our results support that reduced lumbar spine bone mineral density promotes accelerated GrimAge, and perhaps lumbar spine bone mineral density can be a factor affecting Physiological aging. Our findings may provide a promising target for intervention in aging. However, there is a lack of research on the effect of lumbar spine bone mineral density on epigenetic age, so it is hoped that our research will help this new field.

Our study is the first to use Mendelian randomization analysis to explore the causal relationship between epigenetic age and osteoporosis. Mendelian randomization has the advantage that any association between risk factors and disease outcome can be examined by using genetic variants as instrumental variables (IV), which can minimize confusion and avoid reverse causal bias [22, 41]. We selected SNPs with genome-wide associations and independent inheritance but no LD as IV to assess the causal relationship between epigenetic age and osteoporosis. In our analysis, the F-statistic for each SNP was much larger than 10, suggesting that the possibility of weak instrumental variable bias is small. In addition, to ensure the reliability of our results, we identified and removed abnormal IVs by MR-PRESSO outlier testing. We also performed sensitivity analysis to observe whether the results were pleiotropic and heterogeneous.

Our study still has some limitations. Because the pooled GWAS data came from populations of European ancestry, our conclusions may not apply to other ethnic groups. So we use our conclusions with caution in racially and ethnically diverse populations [42]. In this study, GWAS aggregate data was used, and due to the lack of individual data, we could not conduct a stratified analysis of factors such as gender and age. Although every effort was made to exclude confounding in our study, the extent of overlap in exposure and outcome data used in the two-sample MR analysis could not be estimated, and we could only minimize bias in sample overlap by using strong instruments (e.g., F statistic much larger than 10). While we minimized the confounding bias of SNPS, it is still possible that some SNPS are associated with undetected factors that may influence the association between epigenetic age and OP. The level of pleiotropy introduced by this SNP we cannot eliminate its effect on the results. Therefore, the results of MR Analysis should be interpreted with caution.The epigenetic biological GWAS we used are calculated based on blood counts, clinical markers, etc., and may have different results as more samples and GWAS emerge in the future.

## Conclusion

Our findings suggest that there may not be a causal relationship between epigenetic age and osteoporosis, nor is there an association between osteoporosis and changes in epigenetic age. Decreased bone density in the lumbar spine may contribute to the acceleration of GrimAge. Our results are based on the Mendelian randomization analysis we performed. More studies are needed to explore the relationship and potential mechanisms between epigenetic clocks and osteoporosis.

### Supplementary Information

**Additional file 1:** **Supplementary Table 1.** Information on epigenetic age instrumental variables. **Supplementary Table 2.** Information on BMD instrumental variables. **Supplementary Table 3.** Specific information for MR analysis of epigenetic age as exposure and BMD as outcome. ** Supplementary Table 4.** Specific information for MR analysis of BMD as exposure and epigenetic age as outcome.

**Additional file 2:** **Supplementary Figure 1.** “leave-one-out” analyses for MR analysis of epigenetic age as exposure and FN BMD as outcome. (A) GrimAge (B) Hannum (C) HorvathAge (D) PhenoAge. **Supplementary Figure 2.** “leave-one-out” analyses for MR analysis of FN BMD as exposure and epigenetic age as outcome. (A) GrimAge (B) Hannum (C) HorvathAge (D) PhenoAge. **Supplementary Figure 3.** “leave-one-out” analyses for MR analysis of epigenetic age as exposure and FA BMD as outcome. (A) GrimAge (B) Hannum (C) HorvathAge (D) PhenoAge. **Supplementary Figure 4.** “leave-one-out” analyses for MR analysis of FA BMD as exposure and epigenetic age as outcome. (A) GrimAge (B) Hannum (C) HorvathAge (D) PhenoAge. **Supplementary Figure 5.** “leave-one-out” analyses for MR analysis of epigenetic age as exposure and LS BMD as outcome. (A) GrimAge (B) Hannum (C) HorvathAge (D) PhenoAge. **Supplementary Figure 6.** “leave-one-out” analyses for MR analysis of LS BMD as exposure and epigenetic age as outcome. (A) GrimAge (B) Hannum (C) HorvathAge (D) PhenoAge.

## Data Availability

There is no use of raw, unprocessed data in this study. The datasets mentioned in this study can be found in online repositories. The epigenetic age (Intrinsic HorvathAge, HannumAge, PhenoAge and GrimAge) GWAS data source: https://datashare.ed.ac.uk/handle/10283/3645 GWAS summary statistics for bone mineral density (FA BMD, FN BMD and LS BMD): http://www.gefos.org/?q=content/data-release-2015.

## Abbreviations

BMD: Bone mineral density
FN BMD: Femoral neck bone mineral density
LS BMD: Lumbar spine bone mineral density
FA BMD: Forearm bone mineral density
DXA: Dual-energy X-ray absorptiometry
CpG: Cytosine-phosphate-guanine
DNAm: DNA methylation
PAI-1: Plasminogen activation inhibitor 1
MSCs: Mesenchymal stem cells
MR: Mendelian randomization
IV: Instrumental variable
GEFOS: Genetic Factors for Osteoporosis
SNPs: Single nucleotide polymorphisms
kb: Kilobase pairs
LD: Linkage disequilibrium
IVW: Inverse variance weighting
WME: Weighted median method
