# Supplementary material for: Causal association of epigenetic aging and osteoporosis: a bidirectional Mendelian randomization study
Source: BMC Med Genomics. 2023 Nov 2;16:275. doi: 10.1186/s12920-023-01708-3 (PMC10623745; doi:10.1186/s12920-023-01708-3)
Supplement: Supplementary file 1 — Additional file 1: Supplementary Table 1. Information on epigenetic age instrumental variables. Supplementary Table 2. Information on BMD instrumental variables. Supplementary Table 3. Specific information for MR analysis of epigenetic age as exposure and BMD as outcome. Supplementary Table 4. Specific information for MR analysis of BMD as exposure and epigenetic age as outcome. [file 12920_2023_1708_MOESM1_ESM.docx]

| **Supplementary Table 1**. Information on epigenetic age instrumental variables | | | | | | | | | | | |
| --- | --- | --- | --- | --- | --- | --- | --- | --- | --- | --- | --- |
| Exposure | SNP | EA | OA | EAF | Beta | | SE | P value | R^2^ | R^2^ combined | F |
| GrimAge | rs9386796 | T | C | 0.460 | | 0.198 | 0.029 | 1.64E-11 | 0.0195337 | 0.0649438 | 45.49365 |
| GrimAge | rs887466 | A | G | 0.382 | | -0.193 | 0.031 | 5.09E-10 | 0.0175420 |  | 38.68037 |
| GrimAge | rs4065321 | T | C | 0.533 | | -0.170 | 0.030 | 8.78E-09 | 0.0144367 |  | 33.10136 |
| GrimAge | rs17094148 | A | G | 0.707 | | -0.180 | 0.032 | 2.55E-08 | 0.0134314 |  | 31.00560 |
| HannumAge | rs10786282 | A | G | 0.212 | | -0.360 | 0.036 | 2.61E-23 | 0.0433606 | 0.2010492 | 98.89808 |
| HannumAge | rs1005277 | A | C | 0.298 | | 0.301 | 0.033 | 8.92E-20 | 0.0378206 |  | 82.97554 |
| HannumAge | rs12417758 | T | C | 0.546 | | -0.209 | 0.030 | 6.22E-12 | 0.0217170 |  | 47.40141 |
| HannumAge | rs1598856 | A | G | 0.449 | | 0.186 | 0.030 | 3.63E-10 | 0.0170826 |  | 39.40107 |
| HannumAge | rs3093956 | T | C | 0.814 | | -0.243 | 0.040 | 8,39E-10 | 0.0178363 |  | 37.59300 |
| HannumAge | rs4383328 | A | T | 0.285 | | -0.189 | 0.033 | 9.57E-09 | 0.0145366 |  | 32.96645 |
| HannumAge | rs111731678 | A | T | 0.194 | | -0.227 | 0.040 | 1.26E-08 | 0.0160515 |  | 32.41557 |
| HannumAge | rs4838595 | T | C | 0.122 | | -0.258 | 0.045 | 1.29E-08 | 0.0141858 |  | 32.33667 |
| HannumAge | rs34970912 | C | G | 0.965 | | -0.521 | 0.094 | 2.88E-08 | 0.0184580 |  | 30.82083 |
| HorvathAge | rs10949481 | A | T | 0.947 | | 1.082 | 0.070 | 4.56E-54 | 0.1180036 | 0.6745629 | 239.74026 |
| HorvathAge | rs3917672 | A | G | 0.486 | | -0.262 | 0.030 | 5.95E-18 | 0.0343467 |  | 74.39063 |
| HorvathAge | rs57941717 | T | G | 0.253 | | 0.291 | 0.036 | 3.71E-16 | 0.0319472 |  | 66.35175 |
| HorvathAge | rs10447389 | A | G | 0.269 | | -0.276 | 0.034 | 4.42E-16 | 0.0300241 |  | 66.08734 |
| HorvathAge | rs6414374 | A | G | 0.163 | | 0.321 | 0.042 | 1.42E-14 | 0.0281164 |  | 59.12065 |
| HorvathAge | rs4240228 | T | G | 0.717 | | 0.255 | 0.033 | 2.47E-14 | 0.0263378 |  | 58.15204 |
| HorvathAge | rs10732882 | T | G | 0.406 | | -0.241 | 0.032 | 3.68E-14 | 0.0278958 |  | 57.19735 |
| HorvathAge | rs7550821 | T | C | 0.244 | | -0.255 | 0.035 | 3.27E-13 | 0.0240150 |  | 53.12327 |
| HorvathAge | rs7627756 | A | G | 0.562 | | 0.216 | 0.030 | 6.28E-13 | 0.0230176 |  | 51.59153 |
| HorvathAge | rs1511762 | T | C | 0.219 | | 0.264 | 0.037 | 1.53E-12 | 0.0238462 |  | 49.90251 |
| HorvathAge | rs2736099 | A | G | 0.354 | | 0.233 | 0.033 | 3.47E-12 | 0.0247385 |  | 48.49830 |
| HorvathAge | rs12043492 | T | C | 0.421 | | 0.217 | 0.031 | 4.35E-12 | 0.0228751 |  | 47.88817 |
| HorvathAge | rs75243280 | T | C | 0.669 | | -0.232 | 0.034 | 7.37E-12 | 0.0239137 |  | 46.95686 |
| HorvathAge | rs2492286 | T | G | 0.152 | | 0.281 | 0.043 | 5.29E-11 | 0.0203156 |  | 43.04341 |
| HorvathAge | rs6577536 | A | G | 0.484 | | 0.197 | 0.030 | 6.73E-11 | 0.0193460 |  | 42.46551 |
| HorvathAge | rs79111787 | T | C | 0.968 | | -0.908 | 0.141 | 1.28E-10 | 0.0513405 |  | 41.31612 |
| HorvathAge | rs12903325 | T | G | 0.760 | | -0.222 | 0.036 | 5.48E-10 | 0.0179345 |  | 38.53036 |
| HorvathAge | rs1726672 | T | C | 0.304 | | -0.204 | 0.033 | 6.05E-10 | 0.0175630 |  | 38.37219 |
| HorvathAge | rs144317085 | A | T | 0.961 | | 0.514 | 0.083 | 6.42E-10 | 0.0196755 |  | 38.19865 |
| HorvathAge | rs10735418 | T | C | 0.628 | | 0.195 | 0.032 | 1.14E-09 | 0.0178434 |  | 37.05434 |
| HorvathAge | rs1488106 | T | C | 0.372 | | 0.183 | 0.031 | 4.25E-09 | 0.0155976 |  | 34.51090 |
| HorvathAge | rs12666349 | T | C | 0.803 | | 0.255 | 0.044 | 5.23E-09 | 0.0205562 |  | 34.15276 |
| HorvathAge | rs2275558 | A | G | 0.221 | | -0.234 | 0.040 | 6.44E-09 | 0.0187852 |  | 33.73818 |
| HorvathAge | rs34003787 | T | C | 0.086 | | 0.324 | 0.058 | 2.54E-08 | 0.0165276 |  | 31.07919 |
| PhenoAge | rs1142345 | T | C | 0.945 | | 0.824 | 0.087 | 3.35E-21 | 0.0700109 | 0.4277065 | 89.39040 |
| PhenoAge | rs7228835 | C | G | 0.122 | | -0.514 | 0.061 | 3.30E-17 | 0.0565673 |  | 71.11189 |
| PhenoAge | rs678553 | T | C | 0.689 | | 0.327 | 0.041 | 2.27E-15 | 0.0456691 |  | 62.80178 |
| PhenoAge | rs3829957 | T | C | 0.197 | | -0.380 | 0.048 | 3.51E-15 | 0.0456592 |  | 62.02379 |
| PhenoAge | rs752223 | A | G | 0.082 | | -0.560 | 0.072 | 7.20E-15 | 0.0471419 |  | 60.53704 |
| PhenoAge | rs1990053 | A | G | 0.423 | | 0.257 | 0.038 | 2.05E-11 | 0.0323186 |  | 44.89698 |
| PhenoAge | rs11190127 | A | C | 0.379 | | 0.248 | 0.040 | 3.83E-10 | 0.0290325 |  | 39.14913 |
| PhenoAge | rs6531114 | T | C | 0.263 | | -0.254 | 0.042 | 2.07E-09 | 0.0250682 |  | 35.94342 |
| PhenoAge | rs11253338 | T | C | 0.184 | | 0.285 | 0.049 | 8.49E-09 | 0.0243225 |  | 33.19066 |
| PhenoAge | rs116853700 | A | G | 0.043 | | 0.552 | 0.098 | 1.59E-08 | 0.0247721 |  | 31.95261 |
| PhenoAge | rs73028070 | A | G | 0.079 | | -0.433 | 0.077 | 1.74E-08 | 0.0271441 |  | 31.77260 |

| **Supplementary Table 2**. Information on BMD instrumental variables | | | | | | | | | | |
| --- | --- | --- | --- | --- | --- | --- | --- | --- | --- | --- |
| Exposure | SNP | EA | OA | EAF | Beta | SE | P value | R^2^ | R^2^ combined | F |
| FN BMD | rs1366594 | C | A | 0.523 | -0.079 | 0.008 | 5.44E-25 | 0.0031499 | 0.0299808 | 111.48273 |
| FN BMD | rs7524102 | G | A | 0.198 | 0.084 | 0.010 | 7.36E-17 | 0.0022330 |  | 72.77446 |
| FN BMD | rs7108738 | G | T | 0.193 | 0.083 | 0.010 | 8.07E-17 | 0.0021314 |  | 72.58900 |
| FN BMD | rs436448 | T | C | 0.480 | -0.064 | 0.008 | 1.56E-16 | 0.0020349 |  | 71.22557 |
| FN BMD | rs4448201 | G | C | 0.300 | -0.066 | 0.008 | 4.37E-16 | 0.0018077 |  | 69.10568 |
| FN BMD | rs1485307 | T | C | 0.417 | 0.062 | 0.008 | 2.49E-15 | 0.0018453 |  | 65.51637 |
| FN BMD | rs2566752 | C | T | 0.411 | 0.062 | 0.008 | 3.65E-15 | 0.0018573 |  | 64.73152 |
| FN BMD | rs10170839 | C | A | 0.431 | -0.059 | 0.008 | 1.20E-14 | 0.0017285 |  | 62.28842 |
| FN BMD | rs9478217 | A | G | 0.517 | -0.053 | 0.008 | 1.23E-11 | 0.0013889 |  | 48.04778 |
| FN BMD | rs3779381 | G | A | 0.252 | 0.058 | 0.009 | 2.87E-11 | 0.0012683 |  | 46.30290 |
| FN BMD | rs10794639 | G | A | 0.458 | -0.051 | 0.008 | 3.30E-11 | 0.0013010 |  | 46.01535 |
| FN BMD | rs11652763 | A | G | 0.148 | 0.084 | 0.013 | 1.09E-10 | 0.0017570 |  | 43.57940 |
| FN BMD | rs2741856 | C | G | 0.057 | 0.088 | 0.014 | 1.34E-09 | 0.0008304 |  | 38.45650 |
| FN BMD | rs7209460 | C | T | 0.295 | -0.051 | 0.008 | 1.35E-09 | 0.0010741 |  | 38.43274 |
| FN BMD | rs4281029 | A | C | 0.188 | 0.057 | 0.009 | 2.96E-09 | 0.0009846 |  | 36.83807 |
| FN BMD | rs71390846 | C | G | 0.174 | -0.059 | 0.010 | 3.16E-09 | 0.0010027 |  | 36.70452 |
| FN BMD | rs13194508 | C | T | 0.195 | -0.052 | 0.009 | 1.30E-08 | 0.0008453 |  | 33.81719 |
| FN BMD | rs4759320 | C | G | 0.334 | -0.045 | 0.008 | 3.33E-08 | 0.0008935 |  | 31.90904 |
| FN BMD | rs10946458 | C | T | 0.338 | -0.045 | 0.008 | 3.63E-08 | 0.0009035 |  | 31.73134 |
| FN BMD | rs1785493 | T | C | 0.365 | -0.045 | 0.008 | 4.06E-08 | 0.0009435 |  | 31.50202 |
| FA BMD | rs7776725 | A | T | 0.259 | 0.186 | 0.017 | 1.21E-25 | 0.0133064 | 0.0705970 | 114.12705 |
| FA BMD | rs13423976 | A | G | 0.286 | -0.098 | 0.017 | 2.30E-08 | 0.0039206 |  | 32.52830 |
| FA BMD | rs6894139 | C | T | 0.513 | -0.089 | 0.016 | 2.52E-08 | 0.0039521 |  | 32.34466 |
| FA BMD | rs61066067 | T | G | 0.118 | -0.138 | 0.027 | 5.87E-07 | 0.0039638 |  | 25.99227 |
| FA BMD | rs61904896 | C | A | 0.080 | 0.154 | 0.031 | 8.45E-07 | 0.0035058 |  | 25.26202 |
| FA BMD | rs1982763 | A | C | 0.406 | 0.079 | 0.016 | 1.11E-06 | 0.0030234 |  | 24.71887 |
| FA BMD | rs58715565 | G | G | 0.044 | -0.174 | 0.035 | 1.24E-06 | 0.0025789 |  | 24.48849 |
| FA BMD | rs10801580 | A | T | 0.272 | 0.089 | 0.019 | 2.58E-06 | 0.0031302 |  | 23.02342 |
| FA BMD | rs145750983 | C | C | 0.014 | -0.299 | 0.063 | 2.87E-06 | 0.0024247 |  | 22.81310 |
| FA BMD | rs489247 | T | A | 0.319 | -0.086 | 0.018 | 2.90E-06 | 0.0032396 |  | 22.79119 |
| FA BMD | rs1336819 | T | C | 0.063 | -0.234 | 0.049 | 3.17E-06 | 0.0065206 |  | 22.61586 |
| FA BMD | rs35936330 | C | C | 0.168 | 0.095 | 0.020 | 3.30E-06 | 0.0025518 |  | 22.53134 |
| FA BMD | rs7555157 | T | G | 0.280 | 0.084 | 0.018 | 3.75E-06 | 0.0028730 |  | 22.27749 |
| FA BMD | rs7815608 | G | C | 0.270 | -0.081 | 0.017 | 4.34E-06 | 0.0025991 |  | 21.98318 |
| FA BMD | rs7216991 | T | G | 0.459 | -0.074 | 0.016 | 4.87E-06 | 0.0026971 |  | 21.75475 |
| FA BMD | rs115419274 | T | T | 0.026 | 0.451 | 0.097 | 4.90E-06 | 0.0103097 |  | 21.74158 |
| LS BMD | rs9533094 | G | A | 0.404 | -0.083 | 0.009 | 2.80E-20 | 0.0032887 | 0.0397435 | 89.35992 |
| LS BMD | rs2566752 | C | T | 0.411 | 0.083 | 0.009 | 1.49E-19 | 0.0033285 |  | 85.89832 |
| LS BMD | rs7524102 | G | A | 0.198 | 0.090 | 0.011 | 2.41E-14 | 0.0025655 |  | 61.06932 |
| LS BMD | rs7807953 | T | C | 0.263 | 0.075 | 0.010 | 4.11E-14 | 0.0021883 |  | 59.97063 |
| LS BMD | rs2291467 | T | C | 0.228 | -0.077 | 0.010 | 9.64E-14 | 0.0021111 |  | 58.20451 |
| LS BMD | rs1357651 | T | G | 0.355 | -0.068 | 0.009 | 3.75E-13 | 0.0021244 |  | 55.40818 |
| LS BMD | rs1023940 | A | G | 0.465 | 0.065 | 0.009 | 6.47E-13 | 0.0020727 |  | 54.28292 |
| LS BMD | rs894738 | A | G | 0.333 | -0.063 | 0.009 | 2.00E-11 | 0.0017523 |  | 47.22500 |
| LS BMD | rs6965122 | G | A | 0.266 | -0.062 | 0.009 | 7.40E-11 | 0.0014920 |  | 44.53322 |
| LS BMD | rs9749364 | G | T | 0.133 | 0.114 | 0.018 | 6.64E-10 | 0.0030266 |  | 40.03127 |
| LS BMD | rs11002249 | T | C | 0.235 | 0.070 | 0.011 | 1.01E-09 | 0.0017510 |  | 39.18095 |
| LS BMD | rs884205 | A | C | 0.213 | -0.062 | 0.010 | 2.77E-09 | 0.0012982 |  | 37.10351 |
| LS BMD | rs73326583 | A | C | 0.160 | 0.072 | 0.012 | 2.83E-09 | 0.0014103 |  | 37.05857 |
| LS BMD | rs11680288 | G | A | 0.571 | 0.054 | 0.009 | 3.12E-09 | 0.0014410 |  | 36.86079 |
| LS BMD | rs9921222 | T | C | 0.461 | -0.053 | 0.009 | 3.16E-09 | 0.0014143 |  | 36.83906 |
| LS BMD | rs11692564 | T | C | 0.013 | 0.238 | 0.039 | 4.10E-09 | 0.0014215 |  | 36.30283 |
| LS BMD | rs2235811 | G | A | 0.538 | -0.054 | 0.009 | 4.66E-09 | 0.0014659 |  | 36.04114 |
| LS BMD | rs78667121 | A | G | 0.022 | 0.150 | 0.026 | 1.21E-08 | 0.0009748 |  | 34.08780 |
| LS BMD | rs11024028 | G | C | 0.147 | 0.066 | 0.011 | 1.44E-08 | 0.0010861 |  | 33.73327 |
| LS BMD | rs35681117 | T | C | 0.238 | 0.055 | 0.010 | 2.39E-08 | 0.0011035 |  | 32.70962 |
| LS BMD | rs13046645 | A | T | 0.276 | -0.056 | 0.010 | 2.92E-08 | 0.0012480 |  | 32.30203 |
| LS BMD | rs71390846 | C | G | 0.174 | -0.064 | 0.011 | 3.80E-08 | 0.0011790 |  | 31.75832 |

| **Supplementary Table 3**. Specific information for MR analysis of epigenetic age as exposure and BMD as outcome | | | | | | | |
| --- | --- | --- | --- | --- | --- | --- | --- |
| Exposure | Outcome | Methods | Number of IVs | Beta | SE | P value | OR |
| GrimAge | FN BMD | MR Egger | 4 | 0.507 | 0.353 | 0.287 | 1.660(0.832-3.315) |
|  |  | Weighted median | 4 | 0.041 | 0.025 | 0.116 | 1.042(0.990-1.097) |
|  |  | IVW | 4 | 0.022 | 0.021 | 0.297 | 1.023(0.980-1.067) |
|  |  | Simple mode | 4 | 0.044 | 0.036 | 0.327 | 1.045(0.971-1.126) |
|  |  | Weighted mode | 4 | 0.044 | 0.032 | 0.319 | 1.045(0.972-1.124) |
| GrimAge | FA BMD | MR Egger | 4 | -0.433 | 0.735 | 0.615 | 0.649(0.154-2.738) |
|  |  | Weighted median | 4 | -0.009 | 0.056 | 0.867 | 0.991(0.890-1.103) |
|  |  | IVW | 4 | -0.026 | 0.045 | 0.569 | 0.975(0.893-1.064) |
|  |  | Simple mode | 4 | 0.005 | 0.087 | 0.955 | 1.005(0.864-1.169) |
|  |  | Weighted mode | 4 | 0.004 | 0.091 | 0.964 | 1.004(0.849-1.189) |
| GrimAge | LS BMD | MR Egger | 4 | 0.341 | 0.410 | 0.493 | 1.407(0.630-3.140) |
|  |  | Weighted median | 4 | -0.047 | 0.030 | 0.120 | 0.954(0.902-1.010) |
|  |  | IVW | 4 | -0.036 | 0.025 | 0.151 | 0.965(0.919-1.013) |
|  |  | Simple mode | 4 | -0.049 | 0.041 | 0.317 | 0.952(0.881-1.030) |
|  |  | Weighted mode | 4 | -0.049 | 0.039 | 0.296 | 0.952(0.878-1.033) |
| HannumAge | FN BMD | MR Egger | 9 | 0.006 | 0.057 | 0.925 | 1.006(0.899-1.125) |
|  |  | Weighted median | 9 | -0.008 | 0.017 | 0.644 | 0.992(0.959-1.027) |
|  |  | IVW | 9 | 0.002 | 0.015 | 0.915 | 1.002(0.973-1.031) |
|  |  | Simple mode | 9 | -0.008 | 0.029 | 0.781 | 0.992(0.940-1.047) |
|  |  | Weighted mode | 9 | -0.006 | 0.026 | 0.813 | 0.994(0.944-1.046) |
| HannumAge | FA BMD | MR Egger | 9 | 0.057 | 0.091 | 0.553 | 1.059(0.885-1.267) |
|  |  | Weighted median | 9 | 0.040 | 0.035 | 0.253 | 1.041(0.975-1.110) |
|  |  | IVW | 9 | 0.028 | 0.025 | 0.259 | 1.029(0.979-1.081) |
|  |  | Simple mode | 9 | 0.044 | 0.056 | 0.456 | 1.045(0.940-1.162) |
|  |  | Weighted mode | 9 | 0.048 | 0.052 | 0.384 | 1.049(0.949-1.160) |
| HannumAge | LS BMD | MR Egger | 8 | 0.031 | 0.078 | 0.705 | 1.031(0.886-1.201) |
|  |  | Weighted median | 8 | -0.008 | 0.021 | 0.710 | 0.992(0.949-1.037) |
|  |  | IVW | 8 | 0.008 | 0.021 | 0.681 | 1.008(0.969-1.050) |
|  |  | Simple mode | 8 | -0.028 | 0.039 | 0.490 | 0.972(0.903-1.046) |
|  |  | Weighted mode | 8 | -0.027 | 0.034 | 0.452 | 0.974(0.912-1.040) |
| HorvathAge | FN BMD | MR Egger | 24 | 0.013 | 0.019 | 0.521 | 1.013(0.975-1.051) |
|  |  | Weighted median | 24 | -0.005 | 0.011 | 0.628 | 0.995(0.974-1.016) |
|  |  | IVW | 24 | -0.012 | 0.008 | 0.100 | 0.988(0.973-1.002) |
|  |  | Simple mode | 24 | -0.002 | 0.017 | 0.918 | 0.998(0.963-1.034) |
|  |  | Weighted mode | 24 | -0.001 | 0.013 | 0.955 | 0.999(0.975-1.024) |
| HorvathAge | FA BMD | MR Egger | 24 | 0.004 | 0.044 | 0.936 | 1.004(0.920-1.095) |
|  |  | Weighted median | 24 | 0.003 | 0.023 | 0.888 | 1.003(0.962-1.047) |
|  |  | IVW | 24 | -0.009 | 0.017 | 0.579 | 0.991(0.958-1.024) |
|  |  | Simple mode | 24 | 0.032 | 0.039 | 0.410 | 1.033(0.956-1.116) |
|  |  | Weighted mode | 24 | 0.017 | 0.029 | 0.571 | 1.017(0.963-1.074) |
| HorvathAge | LS BMD | MR Egger | 24 | 0.005 | 0.021 | 0.797 | 1.005(0.965-1.047) |
|  |  | Weighted median | 24 | -0.004 | 0.012 | 0.750 | 0.996(0.973-1.020) |
|  |  | IVW | 24 | -0.013 | 0.008 | 0.114 | 0.987(0.972-1.003) |
|  |  | Simple mode | 24 | -0.002 | 0.021 | 0.917 | 0.998(0.960-1.037) |
|  |  | Weighted mode | 24 | -0.002 | 0.015 | 0.912 | 0.998(0.967-1.030) |
| PhenoAge | FN BMD | MR Egger | 11 | 0.011 | 0.024 | 0.667 | 1.011(0.964-1.059) |
|  |  | Weighted median | 11 | -0.003 | 0.011 | 0.812 | 0.997(0.975-1.020) |
|  |  | IVW | 11 | 0.007 | 0.008 | 0.401 | 1.007(0.991-1.023) |
|  |  | Simple mode | 11 | -0.013 | 0.020 | 0.517 | 0.987(0.950-1.025) |
|  |  | Weighted mode | 11 | -0.007 | 0.019 | 0.726 | 0.993(0.958-1.030) |
| PhenoAge | FA BMD | MR Egger | 11 | -0.033 | 0.049 | 0.521 | 0.968(0.879-1.065) |
|  |  | Weighted median | 11 | 0.009 | 0.023 | 0.700 | 1.009(0.967-1.052) |
|  |  | IVW | 11 | 0.011 | 0.017 | 0.534 | 1.011(0.977-1.045) |
|  |  | Simple mode | 11 | 0.044 | 0.038 | 0.270 | 1.045(0.970-1.127) |
|  |  | Weighted mode | 11 | -0.022 | 0.035 | 0.547 | 0.978(0.911-1.051) |
| PhenoAge | LS BMD | MR Egger | 11 | 0.016 | 0.027 | 0.574 | 1.016(0.963-1.072) |
|  |  | Weighted median | 11 | -0.001 | 0.013 | 0.917 | 0.999(0.975-1.023) |
|  |  | IVW | 11 | -0.001 | 0.010 | 0.926 | 0.999(0.980-1.018) |
|  |  | Simple mode | 11 | -0.018 | 0.021 | 0.404 | 0.982(0.943-1.023) |
|  |  | Weighted mode | 11 | 0.014 | 0.020 | 0.489 | 1.014(0.978-1.052) |

| **Supplementary Table 4**. Specific information for MR analysis of BMD as exposure and epigenetic age as outcome | | | | | | | |
| --- | --- | --- | --- | --- | --- | --- | --- |
| Exposure | Outcome | Methods | Number of IVs | Beta | SE | P value | OR |
| FN BMD | GrimAge | MR Egger | 15 | -1.070 | 0.722 | 0.162 | 0.343(0.083-1.412) |
|  |  | Weighted median | 15 | -0.042 | 0.193 | 0.830 | 0.959(0.652-1.413) |
|  |  | IVW | 15 | -0.079 | 0.143 | 0.582 | 0.924(0.699-1.223) |
|  |  | Simple mode | 15 | 0.254 | 0.352 | 0.483 | 1.289(0.646-2.574) |
|  |  | Weighted mode | 15 | -0.468 | 0.323 | 0.170 | 0.626(0.333-1.180) |
| FN BMD | HannumAge | MR Egger | 20 | -0.161 | 0.647 | 0.807 | 0.851(0.239-3.028) |
|  |  | Weighted median | 20 | 0.050 | 0.171 | 0.771 | 1.051(0.750-1.473) |
|  |  | IVW | 20 | 0.172 | 0.125 | 0.169 | 1.188(0.929-1.519) |
|  |  | Simple mode | 20 | 0.062 | 0.307 | 0.842 | 1.064(0.575-1.969) |
|  |  | Weighted mode | 20 | 0.005 | 0.265 | 0.986 | 1.005(0.592-1.707) |
| FN BMD | HorvathAge | MR Egger | 20 | 0.078 | 0.774 | 0.921 | 1.081(0.237-4.927) |
|  |  | Weighted median | 20 | 0.323 | 0.176 | 0.066 | 1.381(0.973-1.962) |
|  |  | IVW | 20 | 0.162 | 0.149 | 0.275 | 1.176(0.879-1.574) |
|  |  | Simple mode | 20 | 0.352 | 0.333 | 0.304 | 1.421(0.717-2.818) |
|  |  | Weighted mode | 20 | 0.388 | 0.291 | 0.199 | 1.474(0.826-2.632) |
| FN BMD | PhenoAge | MR Egger | 20 | -0.325 | 0.928 | 0.730 | 0.723(0.117-4.458) |
|  |  | Weighted median | 20 | 0.250 | 0.219 | 0.253 | 1.284(0.829-1.988) |
|  |  | IVW | 20 | 0.427 | 0.181 | 0.019 | 1.532(1.074-2.186) |
|  |  | Simple mode | 20 | 0.440 | 0.367 | 0.244 | 1.553(0.710-3.398) |
|  |  | Weighted mode | 20 | 0.201 | 0.348 | 0.570 | 1.223(0.626-2.388) |
| FA BMD | GrimAge | MR Egger | 16 | -0.209 | 0.314 | 0.517 | 0.812(0.439-1.501) |
|  |  | Weighted median | 16 | -0.264 | 0.130 | 0.042 | 0.768(0.595-0.990) |
|  |  | IVW | 16 | -0.140 | 0.113 | 0.217 | 0.870(0.697-1.085) |
|  |  | Simple mode | 16 | -0.179 | 0.210 | 0.407 | 0.836(0.542-1.289) |
|  |  | Weighted mode | 16 | -0.310 | 0.165 | 0.079 | 0.733(0.539-0.997) |
| FA BMD | HannumAge | MR Egger | 16 | 0.071 | 0.276 | 0.800 | 1.074(0.626-1.843) |
|  |  | Weighted median | 16 | -0.069 | 0.140 | 0.621 | 0.933(0.714-1.219) |
|  |  | IVW | 16 | -0.059 | 0.101 | 0.557 | 0.943(0.774-1.148) |
|  |  | Simple mode | 16 | -0.198 | 0.222 | 0.387 | 0.821(0.520-1.294) |
|  |  | Weighted mode | 16 | -0.118 | 0.152 | 0.449 | 0.889(0.649-1.216) |
| FA BMD | HorvathAge | MR Egger | 16 | 0.488 | 0.248 | 0.069 | 1.630(1.001-2.653) |
|  |  | Weighted median | 16 | 0.138 | 0.133 | 0.297 | 1.148(0.888-1.486) |
|  |  | IVW | 16 | 0.108 | 0.093 | 0.248 | 1.114(0.928-1.337) |
|  |  | Simple mode | 16 | 0.259 | 0.227 | 0.272 | 1.295(0.841-1.995) |
|  |  | Weighted mode | 16 | 0.178 | 0.170 | 0.310 | 1.195(0.860-1.661) |
| FA BMD | PhenoAge | MR Egger | 16 | 0.163 | 0.320 | 0.619 | 1.177(0.629-2.201) |
|  |  | Weighted median | 16 | 0.042 | 0.162 | 0.794 | 1.043(0.768-1.416) |
|  |  | IVW | 16 | 0.006 | 0.117 | 0.960 | 1.006(0.799-1.266) |
|  |  | Simple mode | 16 | 0.133 | 0.259 | 0.616 | 1.142(0.682-1.911) |
|  |  | Weighted mode | 16 | 0.076 | 0.201 | 0.709 | 1.079(0.718-1.623) |
| LS BMD | GrimAge | MR Egger | 17 | -0.454 | 0.533 | 0.408 | 0.635(0.223-1.806) |
|  |  | Weighted median | 17 | -0.487 | 0.177 | 0.006 | 0.614(0.431-0.875) |
|  |  | IVW | 17 | -0.368 | 0.129 | 0.004 | 0.692(0.538-0.890) |
|  |  | Simple mode | 17 | -0.577 | 0.327 | 0.096 | 0.562(0.307-1.027) |
|  |  | Weighted mode | 17 | -0.605 | 0.308 | 0.067 | 0.546(0.296-1.008) |
| LS BMD | HannumAge | MR Egger | 18 | 0.364 | 0.495 | 0.473 | 1.438(0.545-3.794) |
|  |  | Weighted median | 18 | -0.003 | 0.180 | 0.985 | 0.997(0.701-1.416) |
|  |  | IVW | 18 | -0.058 | 0.128 | 0.650 | 0.944(0.734-1.212) |
|  |  | Simple mode | 18 | -0.390 | 0.385 | 0.326 | 0.677(0.317-1.448) |
|  |  | Weighted mode | 18 | -0.443 | 0.388 | 0.269 | 0.642(0.292-1.410) |
| LS BMD | HorvathAge | MR Egger | 22 | 0.363 | 0.569 | 0.531 | 1.437(0.471-4.380) |
|  |  | Weighted median | 22 | 0.339 | 0.170 | 0.046 | 1.404(1.026-1.921) |
|  |  | IVW | 22 | 0.088 | 0.132 | 0.503 | 1.092(0.844-1.415) |
|  |  | Simple mode | 22 | 0.418 | 0.307 | 0.187 | 1.519(0.836-2.760) |
|  |  | Weighted mode | 22 | 0.432 | 0.305 | 0.171 | 1.541(0.821-2.892) |
| LS BMD | PhenoAge | MR Egger | 22 | 0.180 | 0.700 | 0.800 | 1.197(0.304-4.717) |
|  |  | Weighted median | 22 | -0.064 | 0.206 | 0.757 | 0.938(0.612-1.438) |
|  |  | IVW | 22 | -0.060 | 0.157 | 0.704 | 0.942(0.692-1.282) |
|  |  | Simple mode | 22 | -0.501 | 0.501 | 0.329 | 0.606(0.228-1.611) |
|  |  | Weighted mode | 22 | -0.606 | 0.535 | 0.270 | 0.546(0.202-1.471) |
