# Supplementary material for: Causal association of epigenetic aging and osteoporosis: a bidirectional Mendelian randomization study
Source: BMC Med Genomics. 2023 Nov 2;16:275. doi: 10.1186/s12920-023-01708-3 (PMC10623745; doi:10.1186/s12920-023-01708-3)

**Supplementary Figure 1.** “leave-one-out” analyses for MR analysis of epigenetic age as exposure and FN BMD as outcome. (A) GrimAge (B) Hannum (C) HorvathAge (D) PhenoAge


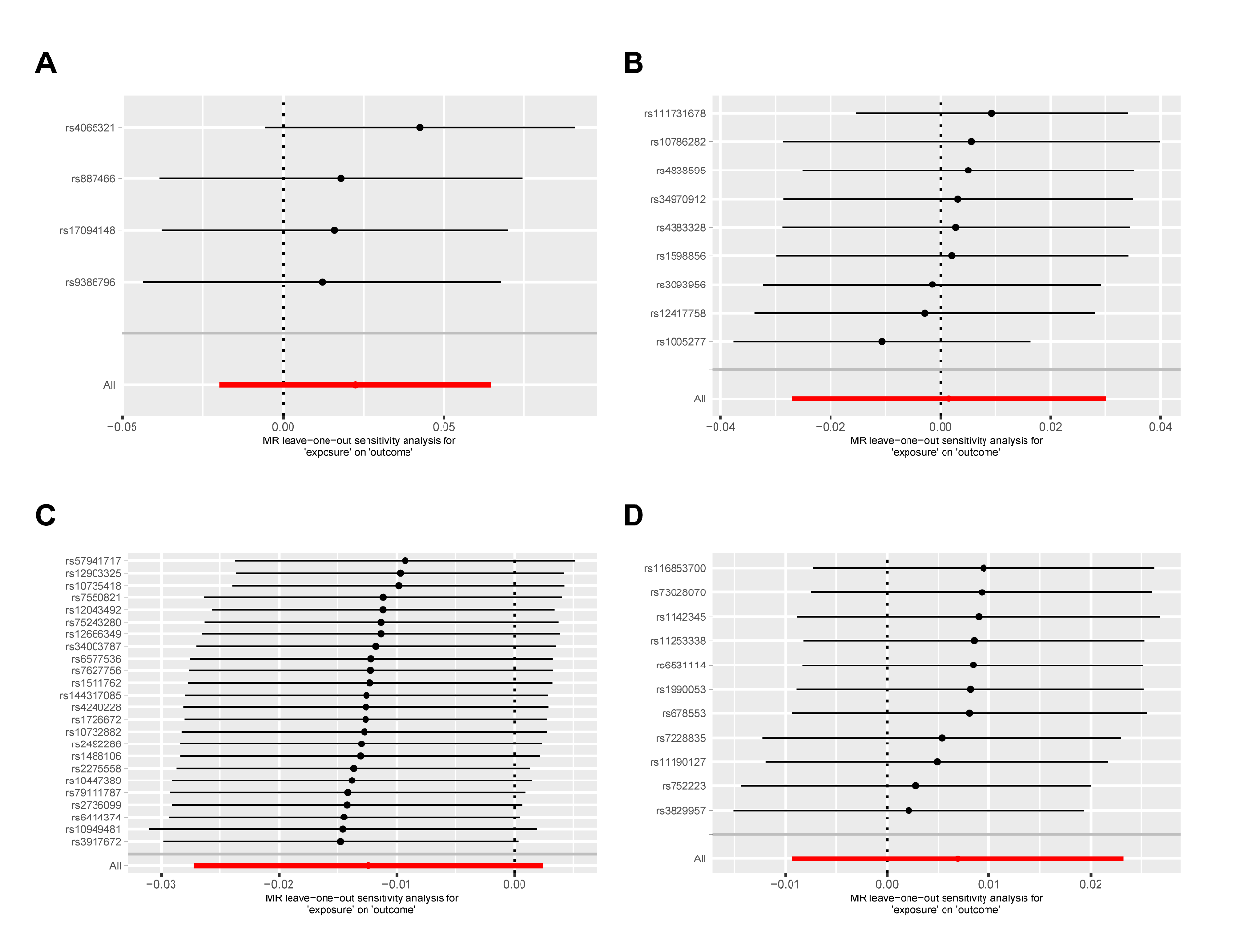


**Supplementary Figure 2.** “leave-one-out” analyses for MR analysis of FN BMD as exposure and epigenetic age as outcome. (A) GrimAge (B) Hannum (C) HorvathAge (D) PhenoAge


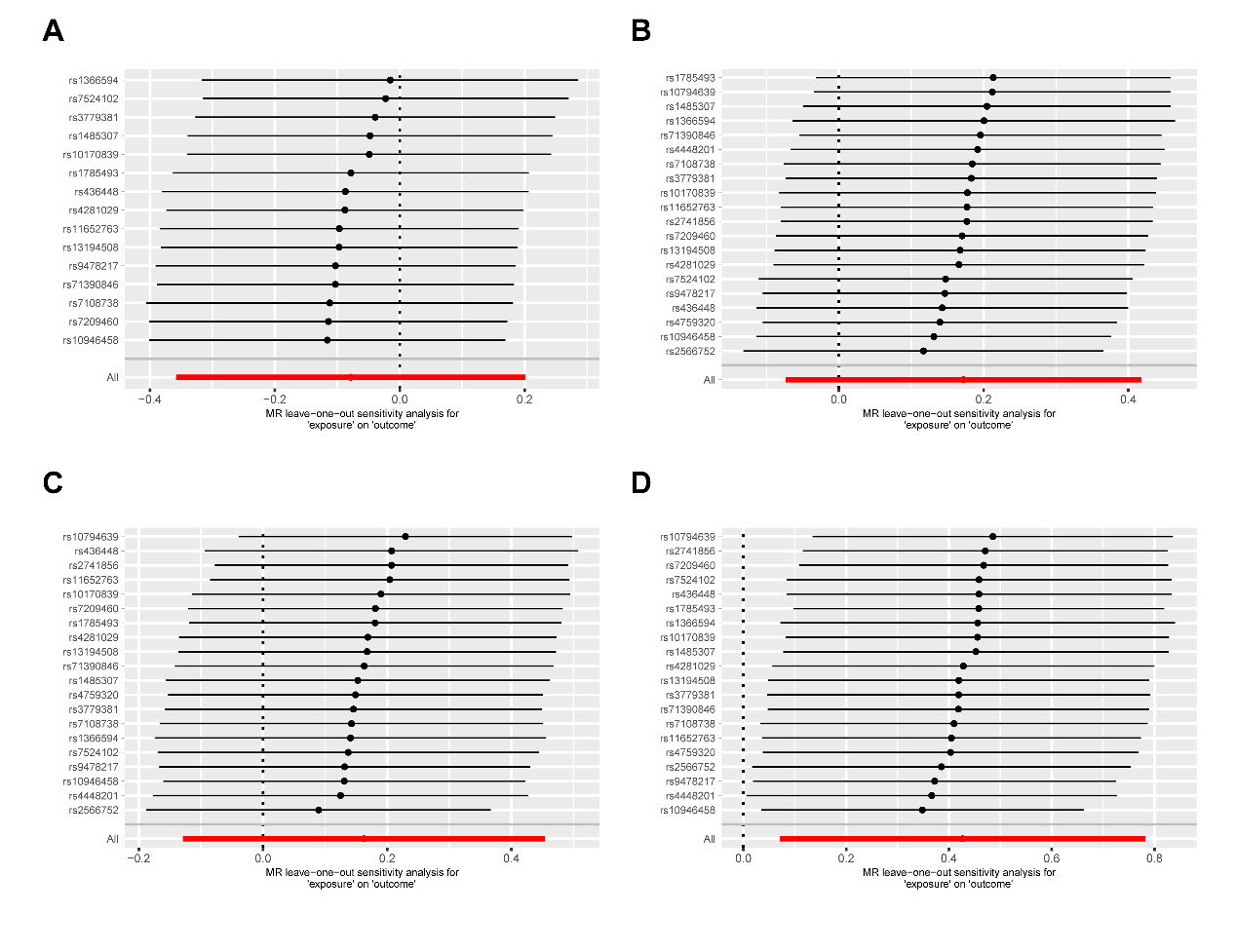


**Supplementary Figure 3.** “leave-one-out” analyses for MR analysis of epigenetic age as exposure and FA BMD as outcome. (A) GrimAge (B) Hannum (C) HorvathAge (D) PhenoAge


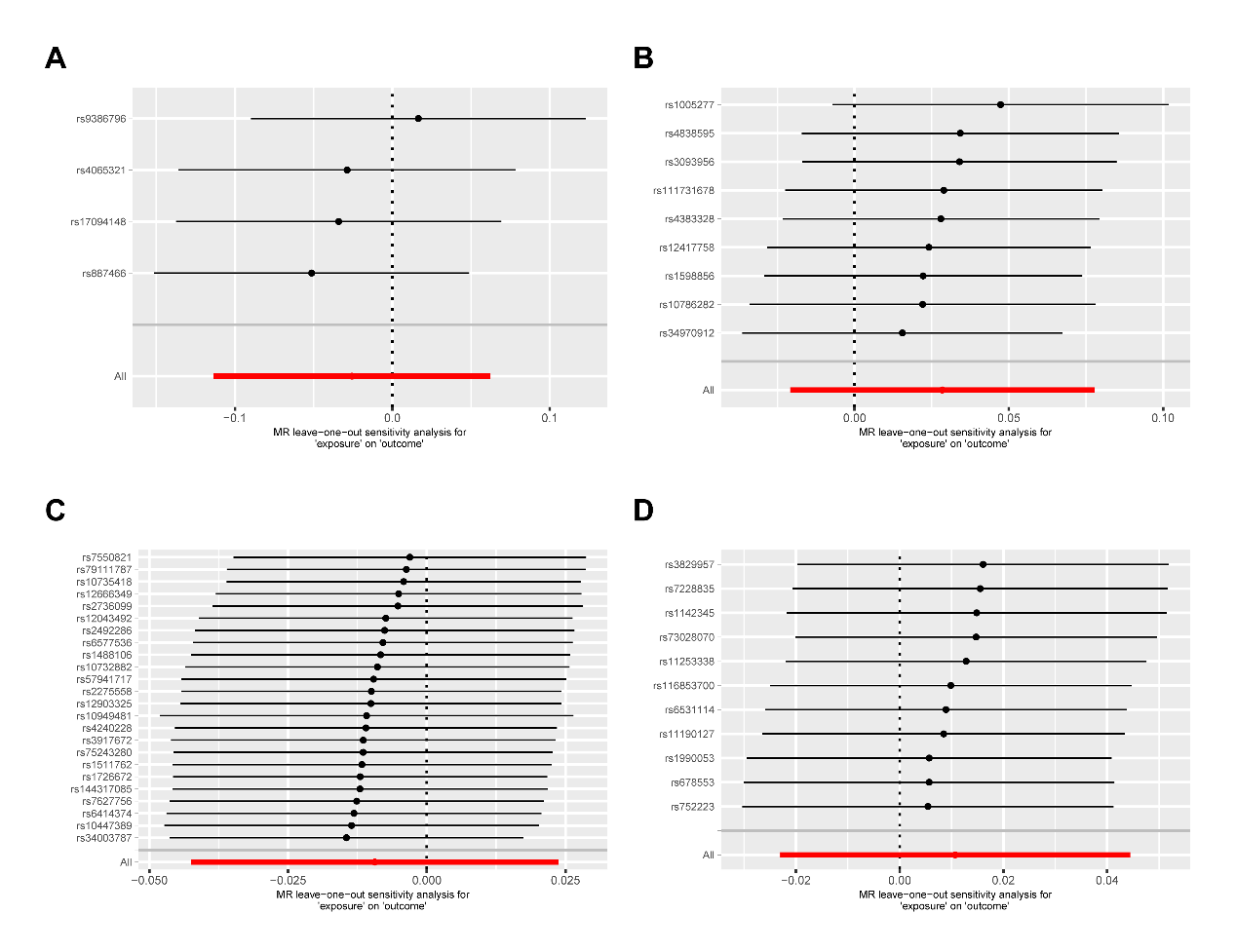


**Supplementary Figure 4.** “leave-one-out” analyses for MR analysis of FA BMD as exposure and epigenetic age as outcome. (A) GrimAge (B) Hannum (C) HorvathAge (D) PhenoAge


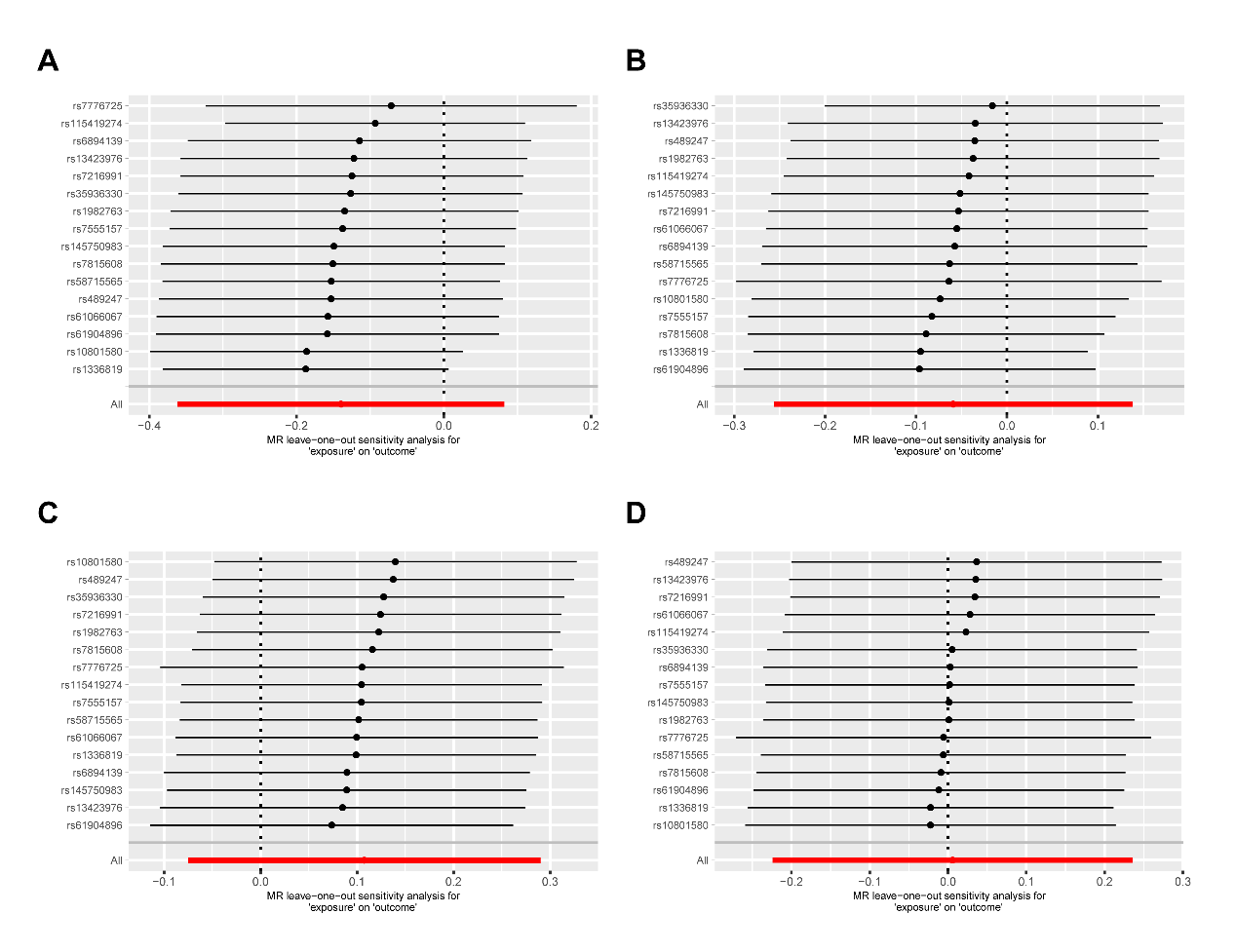


**Supplementary Figure 5.** “leave-one-out” analyses for MR analysis of epigenetic age as exposure and LS BMD as outcome. (A) GrimAge (B) Hannum (C) HorvathAge (D) PhenoAge


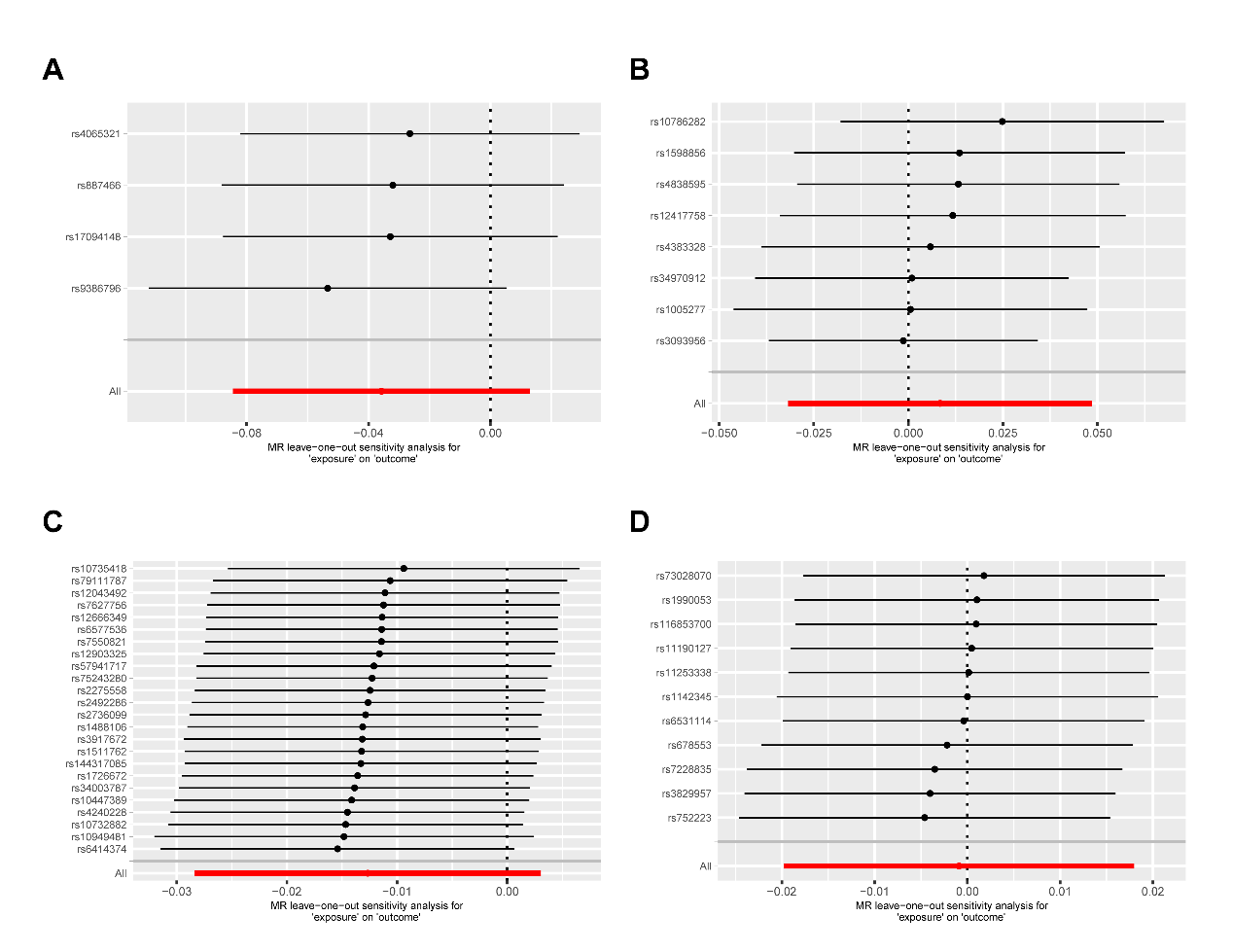


**Supplementary Figure 6.** “leave-one-out” analyses for MR analysis of LS BMD as exposure and epigenetic age as outcome. (A) GrimAge (B) Hannum (C) HorvathAge (D) PhenoAge


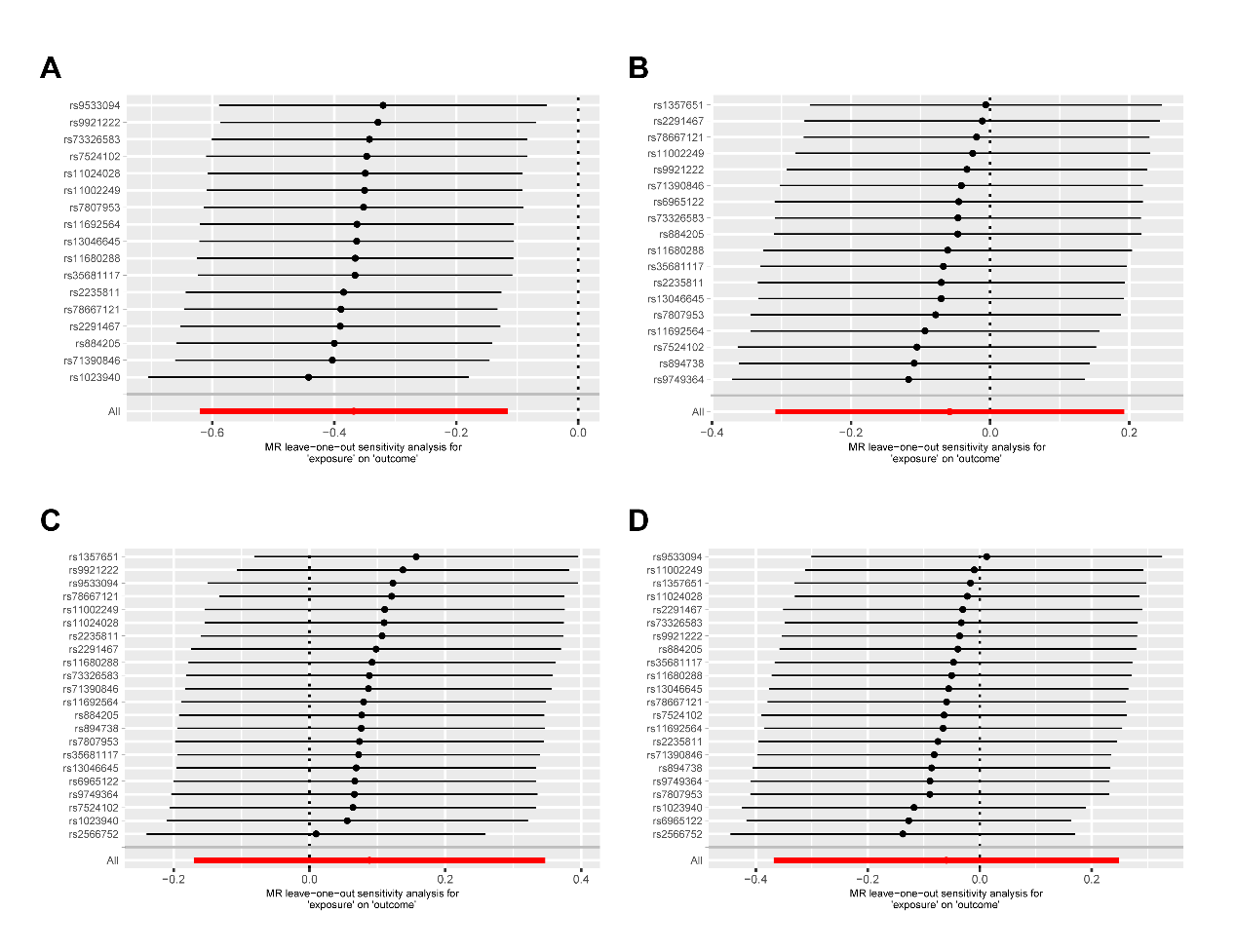

Supplement: Supplementary file 2 — Additional file 2: Supplementary Figure 1. “leave-one-out” analyses for MR analysis of epigenetic age as exposure and FN BMD as outcome. (A) GrimAge (B) Hannum (C) HorvathAge (D) PhenoAge. Supplementary Figure 2. “leave-one-out” analyses for MR analysis of FN BMD as exposure and epigenetic age as outcome. (A) GrimAge (B) Hannum (C) HorvathAge (D) PhenoAge. Supplementary Figure 3. “leave-one-out” analyses for MR analysis of epigenetic age as exposure and FA BMD as outcome. (A) GrimAge (B) Hannum (C) HorvathAge (D) PhenoAge. Supplementary Figure 4. “leave-one-out” analyses for MR analysis of FA BMD as exposure and epigenetic age as outcome. (A) GrimAge (B) Hannum (C) HorvathAge (D) PhenoAge. Supplementary Figure 5. “leave-one-out” analyses for MR analysis of epigenetic age as exposure and LS BMD as outcome. (A) GrimAge (B) Hannum (C) HorvathAge (D) PhenoAge. Supplementary Figure 6. “leave-one-out” analyses for MR analysis of LS BMD as exposure and epigenetic age as outcome. (A) GrimAge (B) Hannum (C) HorvathAge (D) PhenoAge. [file 12920_2023_1708_MOESM2_ESM.docx]
